# A Novel Approach to Assess the Predictiveness of a Continuous Biomarker in Early Phases of Drug Development

**DOI:** 10.1002/sim.70026

**Published:** 2025-02-25

**Authors:** Alessandra Serra, Julia Geronimi, Sandrine Guilleminot, Hugo Hadjur, Marie‐Karelle Riviere, Gaëlle Saint‐Hilary, Pavel Mozgunov

**Affiliations:** ^1^ University of Cambridge MRC Biostatistics Unit Cambridge UK; ^2^ Institut de Recherches Internationales Servier Gif‐sur‐Yvette France; ^3^ Department of Statistical Methodology Saryga Tournus France

**Keywords:** continuous biomarker, early phase, personalized medicine, predictive

## Abstract

Identifying and quantifying predictive biomarkers is a critical issue of personalized medicine approaches and patient‐centric clinical development strategies. In early stages of the development process, significant challenges and numerous uncertainties arise. One of the challenges is the ability to assess the predictive value of a biomarker, i.e., the difference in primary outcomes between experimental and placebo arms above and below a certain threshold of the biomarker. Indeed, when the accumulated information is very limited and the sample size is small, preliminary conclusions about the predictive properties of the biomarker might be misleading. To date, the majority of investigations regarding the predictiveness of biomarkers were in the setting of moderate‐to‐large sample sizes. In this work, we propose a novel flexible approach inspired by the Kolmogorov‐Smirnov Distance in order to assess the predictiveness of a continuous biomarker in a clinical setting where the sample size is small. Via simulations we show that the proposed method allows to achieve a higher power to declare predictiveness compared to the existing methods under a range of scenarios, whilst still maintaining a control of the type I error at a pre‐specified level.

**Abbreviations**
AUC: area under the curve
BMK: biomarker
ROC: receiver operating characteristics
STEPP: subpopulation treatment effect pattern plot

## 1 Introduction

The advance in understanding the molecular pathology of diseases at the patient level has increased the development of therapies that target particular subclasses of tumors. Biomarkers, which are biological characteristics that can be measured in the setting of a clinical intervention, have been used to drive the drug development process and to optimize and personalize treatment management [1]. These offer the possibility to reduce the size, cost and failure rates of clinical trials [2]. However, it can be challenging to characterize and validate biomarkers, especially in early phases of the drug development, where the sample size is generally relatively small.

When talking about predictive biomarkers, it is important to distinguish them from prognostic biomarkers. A prognostic biomarker provides information about the clinical outcome of a patient regardless of the treatment received, whereas a predictive biomarker affects the effect of the treatment on the outcome. Generally, in order to declare whether the biomarkers could predict a greater benefit, in respect of a clinical outcome, a comparison of the effect of an experimental arm compared to a control is required [3]. Indeed, in single‐arm studies, the prognostic effect can not be distinguished from the predictive effect due to the absence of a control arm, and as a result, the effect on the treatment response with respect to the biomarker (if any) cannot be distinguished from the prognostic effect of the biomarker.

The predictive value of biomarkers has been studied in some single‐arm trials, using summary measures, such as the area under the receiver operating characteristics (ROC) curve, across different potential values of the biomarker measured at baseline [4]. However, analyzing only patients who are administered to the experimental arm can give some insights on how patients with different biomarker values might or might not respond to the treatment, but it is not possible to demonstrate the role (predictive and/or prognostic) of the biomarker.

Structured approaches that can provide directions on how to investigate biomarker properties are needed. In a late phase clinical trial, Gottlow et al. (2019) [5] proposed a structured statistical approach in order to investigate the predictive properties of several potential continuous biomarkers in patients with uncontrolled asthma. The conducted analyses showed that the treatment effect was greatest at high baseline values of two biomarkers and thus these were declared to be potentially predictive of the treatment response. These analyses were mainly exploratory and no formal procedure on how to control the type I error rate was mentioned.

Various methods have been proposed in the literature for establishing the predictive properties of a biomarker in a two‐arm setting, where a clinical outcome for patients receiving an experimental treatment is compared with those receiving a control or a placebo treatment. Methodologies for comparing two ROC curves have also been proposed by DeLong et al. (1988) [6] and Blangero et al. (2020) [7]. An alternative commonly used technique to evaluate a predictive biomarker is to model the relationship between outcome, treatment and biomarker through a regression model, and then perform a statistical test for the interaction term between biomarker and treatment [8, 9]. However, this approach can result in a low power as it has been shown that sample size required for detecting interactions needs to be much larger than for detecting main effects, by a factor of up to 16 [10]. Another common approach is to evaluate the treatment effect by biomarker subgroups, but this approach may lead to erroneous conclusions, for example, in the case where the same treatment effect is observed in both biomarker subgroups but the effect is significant only in one of them [9].

Pencina et al. (2008) [11] introduced two new measures based on sensitivity and specificity and on reclassification tables and compared them with the area under the curve approach in order to evaluate the association between a biomarker and a clinical outcome. However, further evaluations by Kerr et al. (2011) [12] concluded that the likelihood ratio test applied in a setting of a regression model is more powerful than the other methods.

All the existing methods used to assess a potential predictive effect of the biomarker have been validated in high‐dimensional data, and their application in small sample size studies requires further development and exploration. Indeed, when the sample size is small several challenges can arise. The variability in the observed data can be very large and commonly used methods such as the standard biomarker‐by‐treatment interaction test might not detect a potential true interaction effect. In addition, when regression models are used, convergence issues may rise if the sample size is too small and especially if the complexity of the model increases (e.g., inclusion of the interaction terms or non‐linear deficiencies).

The objective of this work is two‐fold. Firstly, we propose a novel parametric approach that consists of modeling the relationship between a clinical outcome and a biomarker with the use of a regression model and computing the average difference between the estimated treatment effect (experimental vs. control) for different values of the biomarker. Secondly, we investigate the operating characteristics of the novel approach and other existing methods in the setting where the sample size is limited. A structured procedure on how to control the false positive conclusions at a pre‐specified level and under different configurations and distributions of the biomarker is also described in this work.

## 2 Motivating Example

This work is motivated by a Phase 1/2 clinical trial in patients with advanced Non‐Small Cell Lung Cancer (NSCLC) conducted by Servier. In cancer immune therapy, the role of an enzyme promoting immune suppression in the tumor environment measured at baseline has been investigated as a potential predictive biomarker for the overall response to treatment.

The patients in the study will be randomized in a 2:1 ratio (in favour of the experimental) with the total sample size of 60 patients. The primary objective of the study is to evaluate how the treatment effect of the experimental arm is measured compared to the control regardless of the biomarker values. However, when considering the objective of the analysis of the potential predictive effect of the biomarker, several challenges arise. The sample size is small, and it is unbalanced with more patients allocated to the experimental arm. Moreover, the distribution of the biomarker is unknown and the biomarker might have a prognostic effect. To address these challenges, the following approach is proposed.

## 3 Methodology

In this section, we describe a novel approach inspired by the Kolmogorov‐Smirnov distance, in the setting of the motivating example, to assess whether a biomarker expression has a predictive treatment effect on the overall response rate (ORR).

The Kolmogorov‐Smirnov distance [13, 14] was originally developed to quantify the difference between an empirical distribution ($F_{n}(x)$) of n independent and identically distributed observations of a distribution X (e.g., a biomarker), and a cumulative distribution function (F(x)). This is defined as $D_{n}=\sup_{x\in X}|F_{n}(x)-F(x)|$ and it considers the maximum difference between two distributions across all values of the biomarker.

In this work, we are concerned with the evaluation of the difference between two functions (and the corresponding distribution around them) for different values of the biomarker. Inspired by KS distance, we have considered the maximum difference approach, but this was found to be prone to the random variations in the values of the proposed metrics and hence less robust. Instead, we now propose to consider the average difference between two functions, and hence refer to this as Average Kolmogorov‐Smirnov inspired approach—AKSA.

### 3.1 Setting

Consider a clinical trial with $T_{1}$ being the active treatment and $T_{0}$ a control arm. Assume that a patient's outcome, a response rate, follows a binary distribution, thus $Y^{\left(k\right)}∼Bin(p_{k}),k\in \{0,1\}$ with probability of response $p_{k}$ for the arm k (the control arm is denoted by 0). Consider allocating $n_{1}$ and $n_{0}$ patients to the experimental and control arms respectively, with $N=n_{1}+n_{0}$ being the total sample size. Let's assume to measure a biomarker X that follows a continuous distribution with density function $\phi_{X}(x)$.

### 3.2 Proposed Approach

The proposed procedure is testing whether the treatment effect between experimental and control is the same for different values of the continuous biomarker. The idea of this approach is to consider two biomarker values and their associated difference in responses between treatment and control arm, while taking into account the uncertainty around this difference. The positive difference then implies a possible predictive effect. As the shape of the curve for the difference between treatments may be complex depending on the model used, the procedure repeats this process for different couples of biomarker values to have a global assessment.

Let $f_{k}(x)$ be the true biomarker‐response function on the logit scale for each value x of the biomarker X and for arm k∈{0,1}. Define $D_{X}(x)=f_{1}(x)-f_{0}(x)$ to be the difference of the biomarker‐response functions between the experimental and control arms. This depends on the distribution of the biomarker, represented by the index X in $D_{X}(\cdot )$, which is assumed to follow a normal distribution. The null hypothesis of the average difference between treatment effects for different values of the biomarker being negative is tested $H_{0}:{𝔼}_{X}[D_{X}]\leq 0$.

The biomarker‐response relationships $f_{k}(x),x\in X$ is estimated using a regression model $Y∼g(T_{k},X,T_{k}\times X,C)$, with g(.) being a function of the treatment group, the biomarker value (X), the interaction term treatment by biomarker value ($T_{k}\times X$) and C representing other prognostic variables. Then the estimated difference between the biomarker‐response in experimental and control arms is defined as ${\hat{D}}_{X}(x)=g(T_{1},x,T_{1}\times x,C)-g(T_{0},x,T_{0}\times x,C),$ and its standard error $ŝ(x)=\sqrt{se{\left(g(T_{1},x,T_{1}\times x,C)\right)}^{2}+se{\left(g(T_{0},x,T_{0}\times x,C)\right)}^{2}}$. Consider B couples $c_{i}=(x_{1},x_{2}),i\in \{1,\ldots ,B\}$ of two biomarker values $x_{2}$ and $x_{1}$, with $x_{1}=\min\{x_{1},x_{2}\}$, and define $\mu_{i}={\hat{D}}_{X}(x_{2})-{\hat{D}}_{X}(x_{1})$ and its related standard error $\sigma_{i}=\sqrt{ŝ{\left(x_{1}\right)}^{2}+ŝ{\left(x_{2}\right)}^{2}}$. Then let $d_{i}$ be a sampled value from a normal distribution $Z∼N(\mu =\mu_{i},\sigma =\sigma_{i})$. Then $P(D_{X}>0)=\frac{\sum_{i=1}^{B}1_{d_{i}>0}}{B}$, where 1 is the indicator function meaning that $1_{d_{i}>0}=1$ if $d_{i}>0$ and 0 otherwise. The biomarker is declared as predictive if the probability of the average difference is larger than a pre‐specified threshold $\alpha_{AKSA}$, $P(D_{X}>0)>\alpha_{AKSA}$, where $\alpha_{AKSA}$ is defined to control the type I error at level α.

The proposed approach aims to quantify the difference between two response curves and assess the probability of observing a positive treatment effect for two biomarker values, and how confident we are in it. Some theoretical properties of the proposed method are investigated in Section 1 of the Supporting Information and an Algorithm to implement the method is proposed in the same section.

### 3.3 Illustration

**FIGURE 1:**
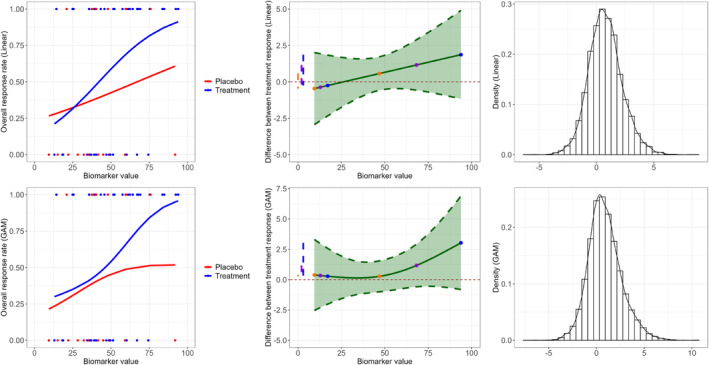
Example of data generated under a hypothetical scenario and corresponding AKSA approach when a linear (top) and a GAM (bottom) models are used to fit the data.

In this section, we provide an illustration example of the proposed approach. In this example, Figure 1, 40 and 20 patients (blue and red points) are generated using a logistic relationship (Section 5.1) between response rate (ORR) and biomarker values for the experimental and control (placebo) arms. For the placebo arm, the response rate is constant across values of the biomarker, thus there is no association between biomarker and response. For the experimental arm instead, the response rate increases sharply with larger values of the biomarker, representing a highly predictive biomarker. We use two models to illustrate the proposed approach, a linear logistic model and a Generalized Additive Model (GAM). The logistic regression model was chosen as it is a natural and standard choice for modeling a binary response outcome. The GAM model was also investigated as it is more flexible and it allows to model also non‐linear shapes that might not be captured by the linear logistic model. This will show the general applicability of the proposed criterion to functions beyond the logistic one.

Firstly, the logistic regression model, Equation (1), is fitted. The estimated curves of response for each treatment arm are represented in the top‐left panel of Figure 1. Then the difference (in the logit scale) between the two fitted curves, ${\hat{D}}_{X}(x)=(({\hat{b}}_{0}+{\hat{b}}_{1}+{\hat{b}}_{2}x+{\hat{b}}_{3}x)-({\hat{b}}_{0}+{\hat{b}}_{3}x))={\hat{b}}_{1}+{\hat{b}}_{2}x$, is represented in the green curve in the middle plot. The dashed lines in green color represent the 95% confidence interval of the difference. The colored points on the green curve on the graph represent some of the randomly sampled couples of biomarker values $\left(x_{1},x_{2}\right)$, while the colored dashed lines on the left represent the corresponding differences ${\hat{D}}_{X}(x_{2})-{\hat{D}}_{X}(x_{1})$. The histogram of the sampled $d_{i},i\in \{1,\ldots ,B\}$ values from the normal distribution with ${\hat{D}}_{X}(x_{2})-{\hat{D}}_{X}(x_{1})$ and standard deviation $\sqrt{ŝ{\left(x_{1}\right)}^{2}+ŝ{\left(x_{2}\right)}^{2}}$ is represented on top‐right panel in Figure 1. The estimated $P(D_{X}>0)$ is of 70%.

In the second case, a GAM is used to fit the data. This is a generalized linear model where the outcome variable depends linearly on some smooth functions (that can be for example polynomials or fractional polynomials [15]) of some predictors. In this case, we model Y, the response variable, as a function of the treatment group and baseline biomarker value as fixed effects, and of z(.), a smoothing term function of the interaction term between baseline biomarker and treatment group, fitted by penalized likelihood using thin plate regression splines [16] with dimension of the basis equal to 3. The model would look like the following: $Y∼T_{k}+X+z(T_{k}\times X)$. The fitted curves for each treatment arm are represented in the bottom‐left panel of Figure 1. Then the difference and its uncertainty between the two fitted curves (in the logit scale) is represented in the green solid and dashed lines respectively in the plot in the middle in Figure 1. The histogram of the sampled $d_{i},i\in \{1,\ldots ,B\}$ values from the normal distribution is represented on a bottom‐right panel in Figure 1. The GAM model is more flexible compared to the linear regression, but in this specific example, it does not seem to improve the fitting of the data compared to the linear logistic regression model (the main differences between the two models can be observed in the fitted curves for the placebo arm). The estimated $P(D_{X}>0)$ is of 69% for GAM.

## 4 Existing Approaches

In this section, we describe alternative approaches used in the literature to assess whether a biomarker is predictive or not.

### 4.1 Interaction Test Approach (IT)

A biomarker is predictive if the treatment effect in the biomarker‐positive patients is different compared to the biomarker‐negative patients. This translates into testing the interaction between the treatment effect and the biomarker value in a regression model. Thus, a conventional analysis for the predictive effect, the test for the interaction biomarker‐by‐treatment term [17] in the generalized linear model, will also be conducted. The generalized linear model with a logit link function (logistic regression) will take the following form: $Y^{\left(k\right)}∼T_{k}+X+T_{k}\times X$, with $Y^{\left(k\right)}$ as response variable, treatment group and biomarker value (X) as fixed effect, and the interaction term treatment by biomarker value ($T_{k}\times X$).

The logistic model will be fitted and the p‐value corresponding to the coefficient of the $T_{k}\times X$ will be compared to $\alpha_{IT}$, which is a threshold specific to this method (and thus the subscript IT) chosen to control the type I error rate at α. If the interaction term hypothesis is rejected, then the biomarker is declared as predictive.

### 4.2 Interaction Test Approach With a Dichotomised Biomarker (ITD)

Motivated by the statistical test used in the biomarker‐driven design proposed by Jiang et al. (2007) [18], we consider a generalized model as described in Section 3 but where the biomarker is considered as a binary variable. In detail, the following procedure is considered:

- the biomarker space is divided into a set of values $\left\{c_{1},c_{2},\ldots ,c_{I}\right\}$;
- for each value, $c_{i}$ with i∈{1,…,I}, a linear logistic model is fitted as following $Y^{\left(k\right)}∼T_{k}+1_{X>c_{i}}+T_{k}\times 1_{X>c_{i}}$, where 1 is the indicator function. The p‐value, $p_{c_{i}}$, for the interaction term ($T_{k}\times 1_{X>c_{i}}$) is computed;
- the minimum p‐value $p_{min}=\min_{c_{i}\in \{c_{1},c_{2},\ldots c_{I}\}}p_{c_{i}}$ for the interaction term is selected and compared to the pre‐specified $\alpha_{ITD}$ level chosen to control the type I error rate at α.

### 4.3 Likelihood Ratio Test (LR)

In this approach, two linear logistic models are compared. One model that does contain only the biomarker and the treatment as covariates, while the second model contains also an additional interaction term between them. The idea is to test whether the full model (the one that contains also the interaction term) fits better the data compared to the simpler model, i.e., test whether adding the interaction term improves or not the fitting. The following two models are compared: $Y^{\left(k\right)}∼T_{k}+X,k\in \{0,1\}$ (Model 1) and $Y^{\left(k\right)}∼T_{k}+X+T_{k}\times X$ (Model 2). Then a likelihood‐ratio test statistics [19] is computed and if the corresponding p‐value is less than a pre‐specified threshold $\alpha_{LR}$, chosen in order to control the type I error rate at α, then the biomarker is declared as predictive.

### 4.4 Subpopulation Treatment Effect Pattern Plot (STEPP) Approach

The Subpopulation Treatment Effect Pattern Plot approach proposed by Yip et al. (2016) [20], consists of exploring the heterogeneity of treatment effects on outcomes across values of continuously measured covariates, such as a biomarker measurement.

The method consists on dividing the population into overlapping subgroups $G_{j},j\in \{1,\ldots ,J\}$ with $r_{2}$ patients in each subgroup and $r_{1}$ maximum number of patients in common among subgroups. Then the treatment effect ${\hat{\theta }}_{j}$ is estimated in each sub‐population and the global null hypothesis of interest is that the treatment effects in all subgroups are the same, that is $H_{0}:\theta_{1}=\cdots =\theta_{J}$. In each subpopulation, a logistic model is fitted on the form of $logit(p_{k})=\beta_{0}+\beta_{1}\times T_{k}$, where $p_{k}$ is probability of response. Then ${\hat{\theta }}_{j}={\hat{p}}_{1,j}-{\hat{p}}_{0,j}$, where ${\hat{p}}_{k,j},k\in \{0,1\},j\in \{1,\ldots ,J\}$ is the estimate of the response rate within the treatment group k inside the sub‐population $G_{j}$. Let ${\hat{\theta }}_{ALL}$ be the estimated overall treatment effect. Then the following test statistics are computed $S=\max_{j=1,\ldots ,J}\left\{\frac{\left|{\hat{\theta }}_{j}-{\hat{\theta }}_{ALL}\right|}{{\hat{\sigma }}_{j}}\right\}$, where ${\hat{\sigma }}_{j}=\sqrt{\hat{var}({\hat{\theta }}_{j}-{\hat{\theta }}_{ALL})}$. A permutation‐based [21] inference is implemented in order to find the final p‐value. Then, if this is below a pre‐specified level $\alpha_{STEPP}$ (chosen in order to control the type I error rate at α), then the global null hypothesis is rejected and we declare the biomarker as predictive as there are some signs of heterogeneity in the population. This approach is implemented in R [22] in the package *stepp* [23] (version 3.2.6).

### 4.5 Probability to Find a Cutoff

This approach consists on declaring that a biomarker is predictive if a cut‐off point is found. In this work, a single cut‐off value for biomarkers is found for both treatment groups. This approach is similar to the common practice of exploring predictiveness by finding one value of the biomarker and then comparing the response rates below and above the selected biomarker value. The cut‐off value will be identified by fitting two step functions with Ordinary Least Squares method, which minimizes the sum of squares considering experimental and control groups separately.

The biomarker cut‐off (and predictive effect) is declared if $P((p_{1a}-p_{0a})-(p_{1b}-p_{0b}))>0)>\alpha_{\text{cutoff}}$ with P being the probability, $p_{1a},p_{1b}$ being the mean response for the experimental arm above and below the cut‐off and $p_{0a},p_{0b}$ the mean for the placebo arm above and below the cut‐off, respectively and $\alpha_{\text{cutoff}}$ is chosen in order to control the type I error rate at α. These responses are assumed to be drawn from a beta distribution with improper prior.

### 4.6 Delong Test to Compare ROC Curves

This approach, proposed by DeLong et al. (1988) [6], consists of comparing the area under the empirical ROC curves (AUC) constructed for the experimental and the control arms.

For each value $c_{k}$ of the biomarker $X_{k}$ and each treatment group k∈K, let define the true positives $TP_{k}$ as the number of cases where $Y^{\left(k\right)}=1$ and $X_{k}<c_{k}$; the false positives $FP_{k}$ as the number of cases where $Y^{\left(k\right)}=0$ and $X_{k}<c_{k}$; the false negatives $FN_{k}$ as the number of cases where $Y^{\left(k\right)}=1$ and $X_{k}\geq c_{k}$ and the true negatives $TN_{k}$ as the number of cases where $Y^{\left(k\right)}=0$ and $X_{k}\geq c_{k}$. Then, for each treatment group a ROC curve, which is a plot of sensitivity versus 1‐specificity, is created with sensitivity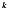 = $\frac{TP_{k}}{TP_{k}+FN_{k}}$ and specitivity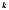 = $\frac{TN_{k}}{TN_{k}+FP_{k}}$. It has been shown that the area under the empirical ROC curve is equal to the Mann‐Whitney two sample statistic [24]. Then, a test to compare the two areas under the curve is proposed by DeLong et al. (1988) [6] and implemented in the package *pROC* [25] (version 1.18.5) in R [22]. The null hypothesis we are interested in is the following: $H_{0}:AUC_{1}-AUC_{0}\leq 0$, where $AUC_{k}$ is the area under the empirical ROC curve for treatment arm k∈{0,1}. Thus, if the resulting p‐value of the test is less than a pre‐specified threshold $\alpha_{DeLong}$ (chosen in order to control the type I error rate at α), then the biomarker is declared as predictive.

## 5 Simulation Study

In this section, we evaluate the performance of the proposed approach using a logistic model and compare it with the competing approaches described in Section 4. The numerical results are found using the software R [22] (version 4.3.0, running under Windows 10 × 64) and 5000 replications (this number of replicates was chosen mainly for computational time constraints as some of the considered methods—e.g., STEPP—might be quite computationally expensive due to multiple permutations of the dataset).

Another approach, the qualitative interaction test, has also been considered but was found to result in low power (see Section 5 of Supporting Information) and hence not included here.

### 5.1 Setting

We consider the clinical trial setting described in Section 2. The primary endpoint is the overall response rate (ORR) and the aim of the study is to explore the association of the biomarker and the clinical response. The overall response rate in the experimental group is assumed to be 60%, while in the control arm 40%.

Data are generated considering a logistic relationship between ORR and biomarker X

(1) $$\begin{array}{ll} p_{k} & =\frac{\exp(b_{0}+b_{1}T_{k}+b_{2}X\times T_{k}+b_{3}X)}{1+\exp(b_{0}+b_{1}T_{k}+b_{2}X\times T_{k}+b_{3}X)}\\ & \;\text{ORR}∼Binom(N,p_{k}) \end{array}$$

where $T_{1}=1$ or $T_{0}=0$ for the experimental or control arms respectively and N is the sample size. The coefficients $b_{i},i\in \{1,\ldots ,3\}$ are chosen to create several scenarios with different degrees of predictive value (H high, M medium, L low, and N none) and with or without a prognostic effect (H high, M moderate, and N none). The difference in response rates between experimental and placebo arms above and below a certain threshold of the biomarker represents the predictive effect of the biomarker. The difference between the response rate above and below a threshold, within the control group, represents the prognostic effect.

For the simulation, predictive effects of 50%, 40%, 30%, 20%, and 0% are considered and the prognostic effect is expressed as a fraction of the predictive ones. The scenarios will be labeled as: NPNPNT non‐predictive non‐prognostic scenario without treatment effect; NPNPT non‐predictive non‐prognostic scenario with treatment effect; NPPNT non‐predictive prognostic scenario without treatment effect; NPPT non‐predictive prognostic scenario with treatment effect; HPNP_50 high predictive non‐prognostic scenario with 50% predictive effect; HPNP_40 high predictive non‐prognostic scenario with 40% predictive effect; MPNP_30 moderate predictive non‐prognostic scenario with 30% predictive effect; LPNP_20 low predictive non‐prognostic scenario with 20% predictive effect.

**FIGURE 2:**
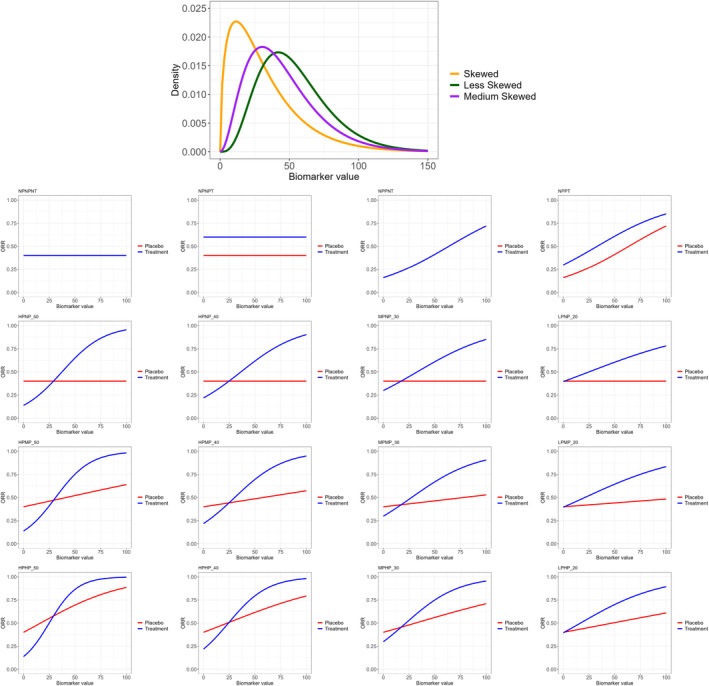
Top‐panel: Representation of the density of the “Skewed”, “Medium skewed” and “Less skewed” gamma distributions for the biomarker X. True null (second‐row) and alternative scenarios (third‐row) from left to right: NPNPNT non‐predictive non‐prognostic scenario without treatment effect; NPNPT non‐predictive non‐prognostic scenario with treatment effect; NPPNT non‐predictive prognostic scenario without treatment effect; NPPT non‐predictive prognostic scenario with treatment effect; HPNP_50 high predictive non‐prognostic scenario with 50% predictive effect; HPNP_40 high predictive non‐prognostic scenario with 40% predictive effect; MPNP_30 moderate predictive non‐prognostic scenario with 30% predictive effect; LPNP_20 low predictive non‐prognostic scenario with 20% predictive effect. True alternative scenarios with moderate prognostic effect (fourth‐row) and high prognostic effect (bottom‐row).

In order to construct these scenarios with different predictive and prognostic effects, a true cutoff for the biomarker needs to be defined. The biomarker is considered to take values between 0 and 100 and the biomarker value that corresponds to the intersection point between the response curves of the control and experimental arms in each scenario is considered to be the true cutoff. For two null scenarios where there is not a predictive effect but there is a prognostic effect (NPPT and NPPNT), the value 17 of the cutoff is chosen in order to have an overall response rate (across all values of the biomarker) of around 40% and 60% for the placebo and experimental arms respectively. The coefficients $b_{i},i\in \{1,\ldots ,3\}$ are given in Table 1 in Section 2 of Supporting Information. The corresponding true response rates of each considered scenario are summarized in Table 2 in Section 2 of Supporting Information. The scenarios with a prognostic effect are constructed using $b_{3}=0.2b_{2}$ or $b_{3}=0.5b_{2}$ for the Moderate and High prognostic cases respectively. The graphical representation of the scenarios is provided in Figure 2.

In the simulation study, different distributions of the biomarker are considered given the uncertainty around this distribution. These are informed by the motivating example described in Section 2 and they reflect the working hypotheses that the biomarker values can range from 0 to 100 and the variance of the biomarker being large. The distributions are represented in Figure 2 and summarized below:

- X∼Γ(r,s), where r is the rate of the Gamma distribution and it can take values of r=0.049 (labeled as “Skewed”), 0.069 (labeled as “Medium skewed”), r=0.083 (labeled as “Less skewed”), and s is the shape parameter that is expressed as $s=650.54r^{2}$ implying the variance of 650.54 informed by the motivating example. The median of X∼Γ(0.083,s) is at 33.29, for X∼Γ(0.049,s) is at 26 and for X∼Γ(0.069,s) is at 40.
- X∼U(a,b), where a=0 and b=100.

The true response rates for all considered scenarios and all types of distributions are summarized in Table 3 in Section 2 of the Supporting Information. The only approach that requires fine‐tuning (apart from the critical value) is the STEPP approach [20] described in Section 4.4. The values $r_{1}=15$ and $r_{2}=40$ were found to result in the highest probability to declare predictiveness (see Section 2.1 of the Supporting Information).

### 5.2 Calibration of the Thresholds

For each method, the pre‐specified thresholds $\alpha_{IT},\alpha_{ITD},$ $\alpha_{LR},\alpha_{STEPP},\alpha_{\text{cutoff}},$ $\alpha_{DeLong},\alpha_{AKSA}$ are chosen in order to control the type I error rate at level α=15% under all the null scenarios across all biomarker distributions.

In particular, for each method, 5000 replications have been performed under the null scenarios (NPNPNT, NPNPT, NPPNT, NPPT) and each distribution of the biomarker described in Section 5.1. For each of those replicates, the resulting p‐value or the probability metric was saved. Then under all these null scenarios, for each method, we compared how many times the saved values were less than a certain threshold. If this proportion of times was less than or equal to 15% for all different distributions of the biomarker, then the corresponding threshold was selected for that specific method.

Thus, for each method, the pre‐specified thresholds are set as: $\alpha_{IT}=0.14$ (Interaction test), $\alpha_{ITD}=0.04$ (Interaction test dichotomized biomarker), $\alpha_{LR}=0.11$ (Likelihood ratio test), $\alpha_{STEPP}=0.13$ (STEPP approach), $\alpha_{\text{cutoff}}=0.997$ (Probability to find a cutoff), $\alpha_{DeLong}=0.15$ (DeLong test), $\alpha_{AKSA}=0.74$ (Difference between two curves).

### 5.3 Results

**FIGURE 3:**
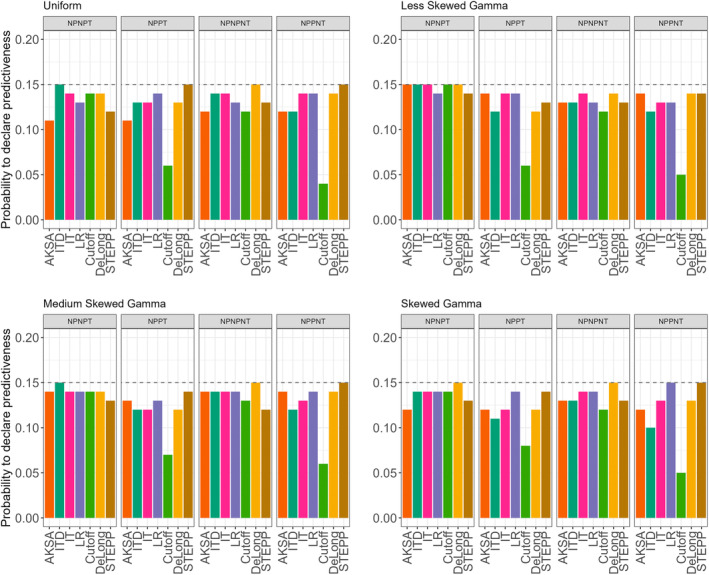
Results for all null scenarios and for all approaches: Interaction test (IT), interaction test with a dichotomous BMK (ITD), likelihood ratio test (LR), STEPP approach (STEPP), probability to find a cutoff (Cutoff), DeLong test (DeLong), difference between two curves (AKSA) for the different distributions of the biomarker and when the sample size is 40:20 patients (experimental:control).

Figure 3 provides the results for all methods and the null scenarios considered in the study. It can be observed that, with the choices of the thresholds as described above, the type I error rate under the null scenarios is below 15% for all methods. However, differences between methods in each scenario can be observed and these are also affected by the distribution of the biomarker. The type I error rate is maximized for AKSA, Interaction tests, and cutoff approaches under the NPNPT when the biomarker has a less skewed gamma distribution. When the biomarker has a medium skewed gamma or uniform distribution, the type I error rate is maximized for the interaction test with a dichotomous BMK under the NPNPT scenario. Overall, it can be observed that the behavior of the different approaches is very different depending on the presence or not of a prognostic effect. Indeed, the presence of the prognostic effect masks the predictive effect of the biomarker and thus it becomes more challenging to detect the predictive value of the biomarker.

**FIGURE 4:**
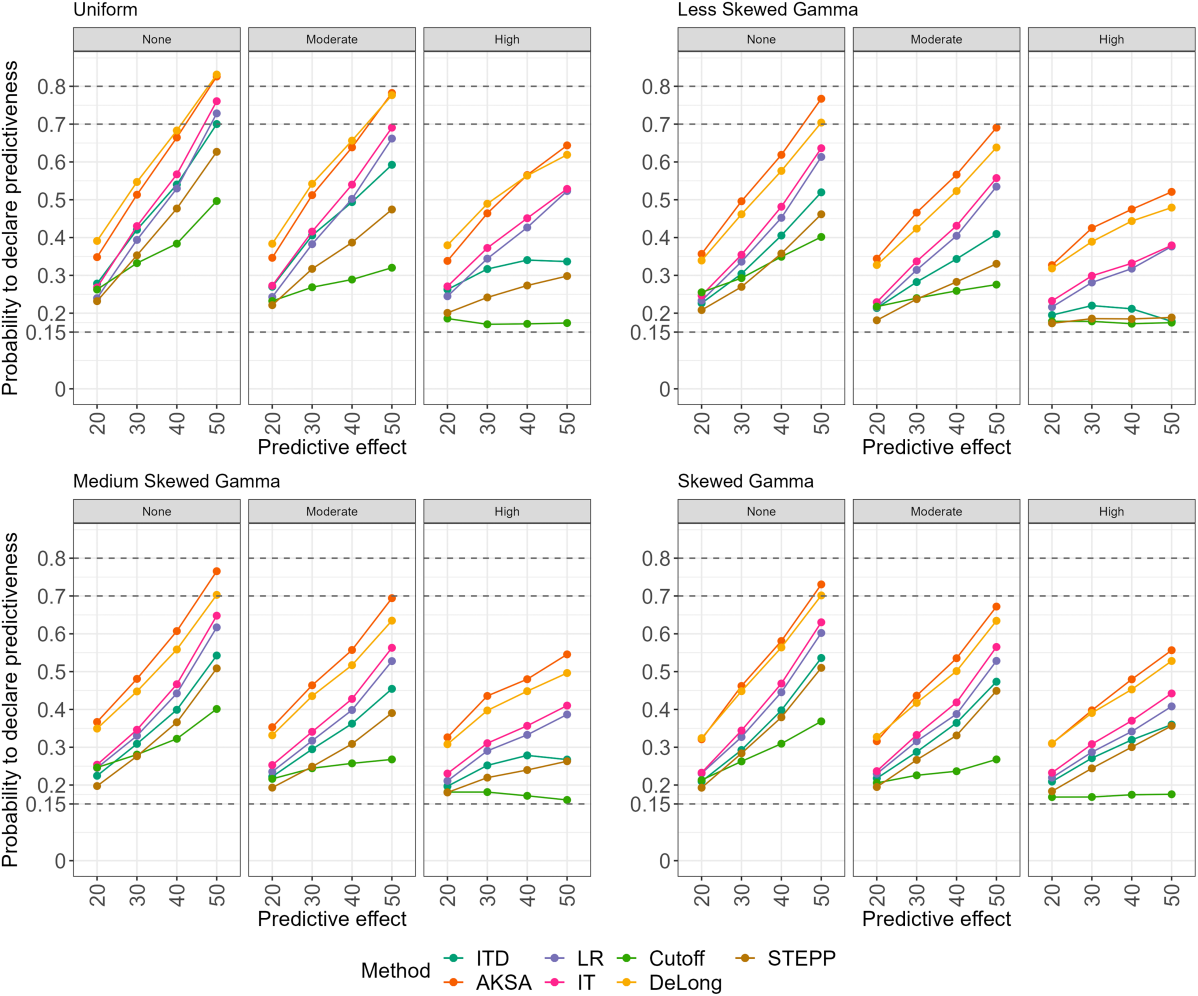
Results for all non‐null scenarios and for all approaches: Interaction test (IT), interaction test with a dichotomous BMK (ITD), likelihood ratio test (LR), STEPP approach (STEPP), probability to find a cutoff (Cutoff), DeLong test (DeLong), difference between two curves (AKSA) for the different distributions of the biomarker and when the sample size is 40:20 patients (experimental:control). Columns in the graphs represent the prognostic effect (N = none, M = moderate and H = high). On the x‐axis, the predictive effect is reported (20%, 30%, 40%, or 50%).

Figure 4 provides the results for all methods and the non‐null scenarios considered in the study. Columns in the graphs represent the prognostic effect (N = null, M = moderate and H = high). On the x‐axis, instead, the predictive effect is reported (20%, 30%, 40%, or 50%). In terms of probability of declaring that the biomarker is predictive, for all non‐null scenarios, the novel approach leads to higher power compared to the other methods.

When the biomarker has a gamma distribution, for AKSA, the power to declare predictiveness ranges between 30% and 77% for the scenarios where the predictive effect is high (i.e., 40% or 50% predictive effect), while it varies between 35% and 70% under the medium predictive effect scenarios (predictive effect of 30%) and between 30% and 50% under the low predictive scenarios (predictive effect of 20%). The DeLong test approach has higher power (up to around 25%) compared to the standard interaction test for all scenarios, while it results in still less power (up to 7%) compared to the proposed approach.

When X∼U(0,100), it can be observed that the novel approach shows similar power compared to the DeLong test in all considered scenarios. Moreover, the proposed method leads to the highest power (it ranges from around 35% up to 83%) for all non‐null scenarios compared to the other distributions of the biomarker. When there is no prognostic effect and the biomarker follows a uniform distribution, the interaction test with continuous biomarker and the likelihood ratio approaches seem to behave quite similarly for different values of the predictive effects. Similarly, the interaction test with a dichotomous biomarker and the STEPP approaches show similar probabilities to declare that the biomarker is predictive for all different levels of predictive effect.

Overall, for all distributions of the biomarker, it can be observed that all other methods result in lower power compared to the standard interaction test approach, with the methods that declare predictiveness once a cutoff is found (method described in Section 4.5) and the STEPP approach (described in Section 4.4) being the least powerful tools (power ranges from just above 15% up to 30% and 70% for the cutoff and STEPP respectively) generally for all scenarios when the predictive effect is high. When a prognostic effect is combined with a predictive effect, then it becomes more challenging to declare that the biomarker is predictive for all methods. Indeed, for each scenario with a medium or high prognostic effect the power is decreased (up to around 20%) compared to the no prognostic cases.

## 6 Sensitivity Analyses

Sensitivity analyses have been conducted to explore the robustness of the proposed method for different sample sizes and for a different relationship between ORR and biomarker. Simulations considering a different data generation mechanism (step function) and with a balanced sample size of 40 or 100 patients in the treatment arms were explored. Results of these analyses can be found in Section 3 of the Supporting Information.

Overall, when data are generated using a step function and the sample size is unbalanced, the type I error rate is controlled at level 15% for almost all methods (slightly inflated in one case) and biomarker scenarios. In general, the results show a much lower (below around 70%) power for all methods, scenarios and distributions of the biomarker compared to the results where the ORR‐biomarker relationship is logistic but with the AKSA and DeLong being overall the best performing approaches.

When a balanced sample size is considered, the type I error rate is controlled at level 15% for all methods (except for the interaction test with binary outcome method where there is an inflation up to 13% and slightly for the IT and AKSA methods in some scenarios) and distributions of the biomarker. In general, the balanced sample size leads to higher power compared to the 40:20 setting for all approaches. The novel approach leads to similar or slightly higher power compared to the DeLong test and to the standard interaction test (up to around 15% when the ORR‐biomarker relationship is logistic). Overall, all other methods lead to a lower power to detect a predictive value of the biomarker compared to these two approaches.

Furthermore, similar patterns and conclusions were drawn for the scenarios where the overall response rate in the experimental groups is equal to 50% (Section 4 of the Supporting Information).

In addition, the operating characteristics of the proposed method and the other considered approaches in the same setting but with a smaller total sample size, compared to the motivating trial, consisting of 30 patients (20 patients in the experimental arm and 10 patients in the control), were also analyzed. Please note that for this specific setting, the parameter $r_{2}$ for the STEPP approach, which controls the maximum number of patients in each subgroup, was set to be equal to 20 and the thresholds for each approach have been re‐calibrated—as described in Section 5.2—in order to control the type I error level at α=15%. Results of these analyses can be found in Section 3.5 of the Supporting Information. When the sample size is smaller than the one in the considered motivating trial, the same overall pattern among methods can be observed. The AKSA method, for all distributions of the biomarker, leads to the highest power (up to roughly 10% compared to DeLong method) to declare predictiveness compared to the other methods and for all considered scenarios. Overall, there is a decrease of roughly 20% in terms of power to declare a predictive effect compared to the setting of the motivating trial.

## 7 Revisiting the Illustration Example

**FIGURE 5:**
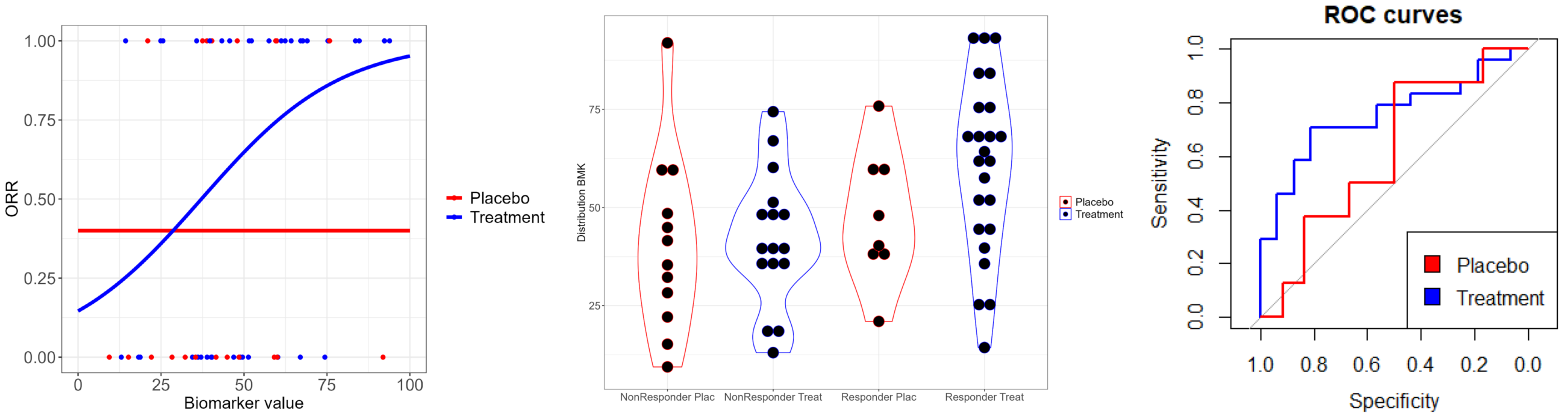
Example of data generated under a hypothetical scenario (left) and distribution of the biomarker per treatment group and outcome (middle). Empirical ROC curves for each treatment group (right).

In the next section, an application of the considered approaches in the setting of the illustration example from Section 3.3 will be presented. The same dataset as before is used and the biomarker values as shown in Figure 5 on the left panel. For this example, the true coefficients $b_{0},b_{1},b_{2},b_{3}$ in the logistic regression in Equation (1) were chosen in order to obtain 40% overall response rate in the placebo group, roughly 80% response rate in the experimental arm for patients with biomarker value above a true cutoff and 40% response rate for patient in the experimental arm below the cutoff. In this case, the biomarker X followed a “Less Skewed” gamma distribution and $\frac{2}{3}$ of the patients were considered to have a biomarker value above the cutoff. On the right panel of Figure 5, the distribution of the biomarker values divided by treatment (experimental and placebo) and outcome groups (responders, Y=1, and non‐responders, Y=0) is represented.

Firstly, we apply the interaction test, Section 4.1. The output of the generalized linear model applied to the generated dataset is summarized in the left panel of Table 1.

**TABLE 1:** Output of the generalized linear model applied to the generated dataset.

IT approach				ITD approach			
Predictors	Log‐Odds	CI	p‐value	Predictors	Log‐Odds	CI	p‐value
(Intercept)	−1.18	−3.70–0.98	0.304	(Intercept)	−0.36	−1.37–0.60	0.469
x	0.02	−0.03–0.07	0.455	I(x > 60)TRUE	−0.34	−3.52–2.19	0.799
trt	−0.72	−3.73–2.36	0.635	trt	0.09	−1.18–1.39	0.884
x:trt	0.03	−0.03–0.09	0.361	I(x > 60)TRUE:trt	2.14	−0.76–5.60	0.161

It can be observed that none of the predictors seem to explain the response rate (all p‐values for all coefficients are above 30%). For the interaction test, we have a corresponding p‐value of 36% and thus if we compare it with the threshold $\alpha_{IT}=0.14$, we are not able to declare that the biomarker has a predictive effect in this case.

The output for the second approach described in Section 4.2 is summarized in the right panel of Table 1. This corresponds to the output of the linear logistic model applied to the dataset when the biomarker is divided into two groups, above and below a value of 60, which corresponds to the cutoff value that provides the minimum p‐value of the interaction term. Here also it can be observed that none of the predictors seem to impact the response and the p‐value of the interaction terms is 16%. If we compare this with the threshold $\alpha_{ITD}=0.04$ then we are not able to declare that the biomarker has a predictive effect.

The likelihood ratio test as described in Section 4.3 provides a p‐value of 37% which means that the additional interaction term does not improve the fitting of the model to the considered data. Moreover, the p‐value is higher than the threshold $\alpha_{LR}=0.11$, and thus we are not able to declare that the biomarker has a predictive effect.

The STEPP method as described in Section 4.4, instead shows some signs of heterogeneity between the treatment groups as the p‐value of 7% is below the selected threshold $\alpha_{STEPP}=0.13$. The probability of finding a cutoff, using the approach described in Section 4.5, is 92% but this is still below the pre‐specified threshold $\alpha_{\text{cutoff}}=0.997$ and thus we are not able to declare that the biomarker has a predictive effect.

The empirical ROC curves constructed for each treatment group, as described in Section 4.6, are represented in the right‐panel of Figure 5. The resulting p‐value from the DeLong test, which compares the area under the two empirical ROC curves, is 19%. Thus, if we compare it with the pre‐specified threshold $\alpha_{DeLong}=0.15$, we are not able to declare that the biomarker is predictive. Finally, the AKSA approach in this specific example provides a probability to declare predictiveness of 70%. This probability is below the pre‐specified $\alpha_{AKSA}=0.74$ value and thus we are not able to declare that the biomarker is predictive.

These results illustrated how methods behave in a specific example. It was observed that the considered methods behave quite differently and provide different answers in a scenario where the biomarker is highly predictive of the treatment response. This is why a simulation study is needed to fully understand the operating characteristics in a more general framework.

## 8 Discussion

The aim of this work was to explore methods that can be used to quantify the predictive value of a continuous biomarker during early phases of the drug development, and thus identify as early as possible potential superior benefits for the patient.

A novel approach AKSA based on the estimated average difference between the biomarker‐response curves in the experimental and control arms has been investigated and compared to standard methods proposed in the literature. The proposed method resulted in higher power (up to 30%) in detecting the predictive value of the biomarker compared to the traditional methods when the sample size is quite limited. Note that with this sample size, the most powerful approach would be able to detect highly predictive biomarkers. This type of approach is therefore particularly well suited to the early identification of BMK showing a marked difference. The proposed approach was based on computing the probability that the average difference between the estimated treatment effect for different values of the biomarker was positive. However, a more marked difference (such as 0.2) could also be chosen. Evaluations of the method for this specific case have been conducted and, as expected, when the marked difference is greater than zero, then it becomes more challenging to detect a larger difference between the two experimental curves.

Overall, for all distributions of the biomarker, it was observed that all approaches (except for AKSA and DeLong) resulted in lower power compared to the standard interaction test approach, with the methods that declare predictiveness once a cutoff is found (method described in Section 4.5) and the STEPP approach (described in Section 4.4) being the least powerful tools generally for all considered scenarios. However, the STEPP approach has the advantage of not being biased by potentially mismodeling the biomarker‐response relationship compared to the other approaches that rely on modeling assumptions. The DeLong and AKSA approaches are the best performing methods. Their performance is similar or even superior for AKSA (up to 7%) when the BMK‐response distribution is skewed.

Limitations in the power to detect a predictive value might also be observed when a prognostic effect of the BMK is present. This is why it is essential to assess the prognostic value of the BMK first, before evaluating the ability of the design to conclude on the predictivity of a BMK. Also, when the sample size is unbalanced, a reduction in the power can be observed. Indeed it has been shown that additional patients in the control arm (i.e., sample size of 40:40 patients) lead to an increase in power for all methods. However, the difference in power between the novel approach and the standard methods is similar to the case when the sample size is not balanced (i.e., 40:20 patients). Having additional patients on the control arm might be beneficial to increase the chance of discovering predictive properties of the biomarker. However, when the sample size is small, relevant historical data coming from previous studies can be used in the analysis.

Finally, it has been observed that it becomes harder to detect a predictive value of the biomarker when the distribution of the response‐biomarker is a step function. This decrease in power, compared to the setting where the relationship of the observed outcome‐biomarker data comes from a logistic function, is expected as data is generated using a step function, while the proposed methods fit the data considering a logistic regression function. Additional explorations would consider how the methods compare when other models are used to fit the data. If multiple regression models are compared, then the model with a better fit of the data (according to some goodness of fit measure such as BIC criterion [26]) can be then selected and a test for declaring the predictive value of the biomarker can be conducted.

In the proposed work, only a binary clinical outcome has been considered. Further extensions of this work include settings where for example the clinical outcome is continuous or a time‐to‐event endpoint. The potential benefits that a continuous outcome instead of a binary one might provide need to be further explored. In addition, a different decision‐making approach, e.g., a “three‐outcome design” with a “consider” zone might be considered in order to limit the probability of stopping the development of the drug when the biomarker can not be declared predictive under the non‐null scenarios. In this case, the idea is to declare that the biomarker is predictive if two conditions are satisfied, when we have a significant chance to show that the average difference between the two groups is positive and a significant chance to show that this is larger than a clinically relevant difference. If none of those conditions are satisfied, then we would declare that the biomarker is not predictive, and if none of the above decisions can be made, then we would consider. This approach has been investigated, but the gain in power was limited compared to the increase in methodological complexity.

The version of the proposed approach (AKSA) can be also further modified in order to ensure that the two biomarker values that are sampled (as explained in Algorithm 1 of the Supporting Information) are not too close to each other, i.e., the couple of biomarker values $\left(x_{1},x_{2}\right)$ must be sampled such as $x_{1}<x_{2}$ and $x_{2}-x_{1}>t$, where t is a pre‐specified value. Indeed if the two points are too close, then their corresponding ${\hat{D}}_{X}(x_{2})-{\hat{D}}_{X}(x_{1})$ will also be close and the uncertainty around can be potentially large. Thus, additional explorations have been done considering a sample strategy where only couples with $x_{2}-x_{1}>2$ were considered in the algorithm, but only minor differences in power (up to around 2% differences) were observed for the considered setting of scenarios.

Overall, the evaluation of the proposed method, compared to the standard methods, is recommended when exploring the predictive value of a continuous biomarker in the early phases of drug development. This study provides a useful tool that can be used, for example, at the end of a Phase II. At this stage, the information on the predictive value of the biomarker can then be taken into account in order to make further recommendations for the next trial (as a Phase III trial). Indeed, if the biomarker shows signs of predictive effect, then methods to identify cutoff values can be explored. Patients can be then stratified by those biomarker values and confirmatory adaptive designs, such as enrichment designs [27], can be implemented in order to further evaluate how the experimental arm can benefit treatment‐sensitive patients.

Several strategies exist for selecting a biomarker threshold. Jiang et al. (2007) [18] proposed a procedure to evaluate the treatment effect in biomarker‐defined subsets in a Phase III trial setting. The approach combines a test for the overall treatment effect in all randomized patients and the validation of a cut point for a prespecified biomarker to identify sensitive patients. However, their proposed procedure might require a large sample size. Other exploratory approaches which investigate the treatment effect in different biomarker‐defined subgroups are proposed by Lipkovich et al. (2011) [28] and Yip et al. (2016) [20]. Lipkovich et al. (2011) [28] proposed a recursive algorithm that consists of partitioning the space of the biomarker into different subgroups such that the treatment effect within each subgroup is maximized. Yip et al. (2016) [20], instead, proposed a method to explore the heterogeneity of the treatment effect in overlapping biomarker‐defined subgroups of patients.

## Author Contributions

All authors have directly participated in the planning and execution of the presented work.

## Conflicts of Interest

J.G. and S.G. are the employees of Institut de Recherches Internationales Servier. G.S.‐H. is President of Saryga SAS. M.‐K.R. and H.H. are employees of Saryga SAS. P.M. and A.S. served as statistical consultants for Institut de Recherches Internationales Servier and Saryga SAS.

## Supporting information




**Data S1.** Additional supporting information may be found online in the Supporting Information section at the end of this article. Programming code for reproducing the numerical results is available at GitHub: https://github.com/aspapercode/predVal.git.
