# Supplementary material for: A Novel Approach to Assess the Predictiveness of a Continuous Biomarker in Early Phases of Drug Development
Source: Stat Med. 2025 Feb 25;44(5):e70026. doi: 10.1002/sim.70026 (PMC11862805; doi:10.1002/sim.70026)
Supplement: Supplementary file 1 — Data S1. Additional supporting information may be found online in the Supporting Information section at the end of this article. Programming code for reproducing the numerical results is available at GitHub: https://github.com/aspapercode/predVal.git. [file SIM-44-0-s001.pdf]

Received: Added at production | Revised: Added at production | Accepted: Added at production
DOI: xxx/xxxx

ARTICLE TYPE

Supplementary material of ‘A novel approach to assess the predictiveness of a continuous biomarker in early phases of drug development’

Alessandra Serra*[1] | Julia Geronimi[3] | Sandrine Guilleminot[3] | Hugo Hadjur[2] | Marie-Karelle Riviere[2] | Gaëlle Saint-Hilary[2] | Pavel Mozgunov[1]

[1]University of Cambridge, MRC Biostatistics Unit, Cambridge, United Kingdom

[2]Department of Statistical Methodology, Saryga, France

[3]Institut de Recherches Internationales Servier, Gif-sur-Yvette, France

Correspondence
*Corresponding author name, This is sample corresponding address.
Email: alessandra.serra@mrc-bsu.cam.ac.uk

Present address
This is sample for present address text this is sample for present address text

Identifying and quantifying predictive biomarkers is a critical issue of personalised medicine approaches and patient-centric clinical development strategies. In early stages of the development process, significant challenges and numerous uncertainties arise. One of the challenges is the ability to assess the predictive value of a biomarker, i.e. the difference in primary outcomes between experimental and placebo arms above and below a certain threshold of the biomarker. Indeed, when the accumulated information is very limited and the sample size is small, preliminary conclusions about the predictive properties of the biomarker might be misleading.
To date, the majority of investigations regarding the predictiveness of biomarkers were in the setting of moderate-to-large sample sizes. In this work, we propose a novel flexible approach inspired by the Kolmogorov-Smirnov Distance in order to assess the predictiveness of a continuous biomarker in a clinical setting where the sample size is small. Via simulations we show that the proposed method allows to achieve a higher power to declare predictiveness compared to the existing methods under a range of scenarios, whilst still maintaining a control of the type I error at a pre-specified level.

KEYWORDS
Predictive, Continuous biomarker, Early Phase, Personalized medicine

Abbreviations: BMK, biomarker; ROC, receiver operating characteristics; AUC, area under the curve; STEPP, Subpopulation Treatment Effect Pattern Plot

 © 2023 Copyright Holder Name

1 | AKSA APPROACH

1.1 | Algorithm

Algorithm 1 AKSA approach

1: Estimate jointly the model for each arm using a maximum likelihood estimator and obtain the estimated biomarker-response $\hat{f}_k(x)$ on the logit scale and its associated standard error $\hat{se}_k(x)$ in each arm $k \in \{0,1\}$.
2: Define for a value x of the biomarker X, $\hat{D}_X(x) = \hat{f}_1(x) - \hat{f}_0(x)$ and its standard error $\hat{s}(x) = \sqrt{\hat{se}_1^2(x) + \hat{se}_0^2(x)}$
3: Sample randomly and with replacement a large number, B, of couples of biomarker values (x_1, x_2) such as $x_1 < x_2$
4: For each sample $c_i = (x_1, x_2), i \in \{1, \ldots, B\}$:
  - Compute $\hat{D}_X(x_2) - \hat{D}_X(x_1)$ and $\sqrt{\hat{s}(x_1)^2 + \hat{s}(x_2)^2}$
  - Sample a value d_i from a normal distribution with mean $\hat{D}_X(x_2) - \hat{D}_X(x_1)$ and standard deviation $\sqrt{\hat{s}(x_1)^2 + \hat{s}(x_2)^2}$.

5: Compute $P(D_X > 0) = \frac{\sum_{i=1}^{B} \mathbb{1}_{d_i > 0}}{B}$
6: If $P(D_X > 0) > \alpha_{AKSA}$, then declare that the biomarker is predictive.

1.2 | Theoretical properties

We consider the clinical trial setting described in Section 2 where the primary endpoint is the overall response rate (ORR). A logistic relationship for the response-biomarker curve is considered, that is:

$$p_k = \frac{\exp(b_0 + b_1 T_k + b_2 X \times T_k + b_3 X)}{1 + \exp(b_0 + b_1 T_k + b_2 X \times T_k + b_3 X)}, \quad Y^{(k)} \sim Binom(N, p_k) \tag{1}$$

where $T_1 = 1$ or $T_0 = 0$ for the experimental or control arms respectively and N is the sample size. Let us consider X to be uniformly distributed between a and b, that is $X \sim (a, b)$.

The logistic regression model is used to fit the data, thus:

$$logit(Y) \sim T_k + X + T_k \times X, \text{ where } logit(Y) = log\left(\frac{Y}{1-Y}\right)$$

Let consider $X_1, X_2 \overset{\text{i.i.d}}{\sim} U(a, b)$ and $X_M = \max\{X_1, X_2\}$, $X_m = \min\{X_1, X_2\}$. Then, let define $f_M(x) = \frac{2(x-a)}{b-a}$ and $f_m(x) = \left(\frac{b-x}{b-a}\right)\left(\frac{2}{b-a}\right)$ to be the densities of X_M and X_m respectively. Let define $D_X(x_2) - D_X(x_1) =$

$$\begin{aligned} &((b_0 + b_1 + b_2 x_2 + b_3 x_2) - (b_0 + b_3 x_2)) \times f_M(x_2) - ((b_0 + b_1 + b_2 x_1 + b_3 x_1) - (b_0 + b_3 x_1)) \times f_m(x_1) \\ &= (b_1 + b_2 x_2) \times f_M(x_2) - (b_1 + b_2 x_1) \times f_m(x_1) \end{aligned}$$

Then let marginalise over all possible values of $x_1 < x_2$, that is

$$d_{Y,b_1,b_2} = \int_a^b \left(\int_{x_1}^b (b_1 + b_2 x_2) \times f_M(x_2) - (b_1 + b_2 x_1) \times f_m(x_1)\, dx_2 \right) dx_1$$

and thus the normalised mean of the average difference is $\mu_{D_X,b_1,b_2} = \frac{d_{Y,b_1,b_2}}{b-a}$. However, also the standard error of the average difference (coming from the standard error of the estimated coefficients of the logistic regression) needs to be taken into account. Thus, the same average difference is also computed considering the coefficients of the logistic regression being one standard

deviation away from the mean, that is $\mu_{D_X,b_1-se_{b_1},b_2-se_{b_2}}$. The standard errors of the coefficients, $se_{b_i}, i \in \{0,\ldots,3\}$ are computed following the equation in Section 5.5.2 of Agresti (2002)[1].

To compute the standard error, define for the experimental arm:

$$d_{M1} = \int_a^b (b_0 + b_1 + b_2x + b_3x) \times f_M(x)\,dx \ \text{ and } \ d_{m1} = \int_a^b (b_0 + b_1 + b_2x + b_3x) \times f_m(x)\,dx$$

then transform these on the probability scale:

$$p_{M1} = \frac{1}{1+\exp(-d_{M1})} \ \text{ and } \ p_{m1} = \frac{1}{1+\exp(-d_{m1})}$$

and define $p_{c1} = \frac{1}{1+\exp(-(b_0+b_1))}$. Then, using the delta method[2] applied to the logit function transformation, the variance on the logit scale for the experimental arm is computed as:

$$se_1^2 = \frac{p_{M1}\times(1-p_{M1})\times\left(\frac{1}{p_{M1}}+\frac{1}{1-p_{M1}}\right)^2+p_{m1}\times(1-p_{m1})\times\left(\frac{1}{p_{m1}}+\frac{1}{1-p_{m1}}\right)^2+2\times p_{c1}\times(1-p_{c1})\times\left(\frac{1}{p_{c1}}+\frac{1}{1-p_{c1}}\right)^2}{n_1}$$

Similarly, the standard error is computed for the control arm with:

$$d_{M0} = \int_a^b (b_0 + b_3x) \times f_M(x)\,dx \ \text{ and } \ d_{m0} = \int_a^b (b_0 + b_3x) \times f_m(x)\,dx$$

then let us transform these on the probability scale:

$$p_{M0} = \frac{1}{1+\exp(-d_{M0})} \ \text{ and } \ p_{m0} = \frac{1}{1+\exp(-d_{m0})}$$

and define $p_{c0} = \frac{1}{1+\exp(-(b_0))}$. Then, the variance on the logit scale for the control arm is:

$$se_0^2 = \frac{p_{M0}\times(1-p_{M0})\times\left(\frac{1}{p_{M0}}+\frac{1}{1-p_{M0}}\right)^2+p_{m0}\times(1-p_{m0})\times\left(\frac{1}{p_{m0}}+\frac{1}{1-p_{m0}}\right)^2+2\times p_{c0}\times(1-p_{c0})\times\left(\frac{1}{p_{c0}}+\frac{1}{1-p_{c0}}\right)^2}{n_0}$$

and thus the total standard error is

$$s_{D_X} = \sqrt{se_1^2 + se_0^2}$$

Thus, $P(P(D_X > 0) > \alpha_{AKSA})$ can be approximated by

$$P(P(D_X > 0) > \alpha_{AKSA}) = P(Z < \epsilon)$$

where

$$Z \sim N(\mu = \mu_{D_X,b_1,b_2} - \mu_{D_X,b_1-se_{b_1},b_2-se_{b_2}}, \sigma = s_{D_X})$$

and $\epsilon = -\Phi^{-1}(\alpha_{AKSA})$, that is the quantile of a standard normal distribution evaluated in α_{AKSA}.

2 | SCENARIOS FOR SIMULATION STUDY

Coefficients (term)	NPNPT	NPPT	NPNPNT	NPPNT	HPNP_50	HPNP_40	MPNP_30	LPNP_20
b_0	-0.405	-1.655	-0.405	-1.655	-0.405	-0.405	-0.405	-0.405
b_1	0.811	0.8	0	0	-1.428	-0.868	-0.45	-0.02
b_2	0	0	0	0	0.049	0.035	0.026	0.017
b_3	0	0.026	0	0.026	0	0	0	0
True cutoff	-	17	-	17	30	26	17	1

TABLE 1 True coefficients and true cutoff values for the logistic regression model for null scenarios and the alternative scenarios without a prognostic effect.

	Cutoff biomarker	Placebo			Experimental		
		Rate Below cut-off	Rate Above cut-off	Overall Rate	Rate Below cut-off	Rate Above cut-off	Overall Rate
NPNPT	NA	NA	NA	0.4	NA	NA	0.6
NPPT	17	0.19	0.47	0.42	0.35	0.65	0.6
NPNPNT	NA	NA	NA	0.4	NA	NA	0.4
NPPNT	17	0.19	0.47	0.42	0.19	0.47	0.42
HPNP_50	30	0.4	0.4	0.4	0.26	0.75	0.6
HPNP_40	26	0.4	0.4	0.4	0.31	0.7	0.6
MPNP_30	17	0.4	0.4	0.4	0.35	0.65	0.6
LPNP_20	1	0.4	0.4	0.4	0.4	0.6	0.6

TABLE 2 True response rates above and below a biomarker cutoff for the two treatment groups and for the following scenarios: Non-Predictive Non-Prognostic scenario without treatment effect (NPNPNT), Non-Predictive Non-Prognostic scenario with treatment effect (NPNPT), Non-Predictive Prognostic scenario with treatment effect (NPPT), Non-Predictive Prognostic scenario without treatment effect (NPPNT), High Predictive No Prognostic scenario (HPNP_50), High Predictive No Prognostic scenario (HPNP_40), Medium Predictive No Prognostic scenario (MPNP_30), Low Predictive No Prognostic scenario (LPNP_20)

	$X \sim U(0, 100)$						$X \sim \Gamma(0.049, s)$						$X \sim \Gamma(0.083, s)$						$X \sim \Gamma(0.069, s)$					
Scenario	Placebo			Experimental			Placebo			Experimental			Placebo			Experimental			Placebo			Experimental		
	Rate Below cut-off	Rate Above cut-off	Overall Rate	Rate Below cut-off	Rate Above cut-off	Overall Rate	Rate Below cut-off	Rate Above cut-off	Overall Rate	Rate Below cut-off	Rate Above cut-off	Overall Rate	Rate Below cut-off	Rate Above cut-off	Overall Rate	Rate Below cut-off	Rate Above cut-off	Overall Rate	Rate Below cut-off	Rate Above cut-off	Overall Rate	Rate Below cut-off	Rate Above cut-off	Overall Rate
NPNPT	NA	NA	0.4	NA	NA	0.6	NA	NA	0.4	NA	NA	0.6	NA	NA	0.4	NA	NA	0.6	NA	NA	0.4	NA	NA	0.6
NPPT	0.19	0.47	0.42	0.35	0.65	0.6	0.2	0.37	0.32	0.35	0.56	0.49	0.21	0.45	0.44	0.38	0.63	0.62	0.21	0.41	0.39	0.37	0.59	0.57
NPNPNT	NA	NA	0.4	NA	NA	0.4	NA	NA	0.4	NA	NA	0.4	NA	NA	0.4	NA	NA	0.4	NA	NA	0.4	NA	NA	0.4
NPPNT	0.19	0.47	0.42	0.19	0.47	0.42	0.2	0.37	0.32	0.2	0.37	0.32	0.21	0.45	0.44	0.21	0.45	0.44	0.21	0.41	0.39	0.21	0.41	0.39
HPNP_50	0.4	0.4	0.4	0.26	0.75	0.6	0.4	0.4	0.4	0.26	0.65	0.43	0.4	0.4	0.4	0.33	0.7	0.64	0.4	0.4	0.4	0.3	0.67	0.55
HPMP_50	0.44	0.56	0.52	0.29	0.83	0.67	0.44	0.53	0.48	0.29	0.74	0.48	0.45	0.55	0.53	0.38	0.78	0.72	0.45	0.54	0.51	0.35	0.76	0.62
HPHP_50	0.49	0.76	0.68	0.34	0.9	0.73	0.49	0.7	0.58	0.34	0.83	0.55	0.54	0.73	0.7	0.46	0.87	0.8	0.52	0.71	0.65	0.42	0.85	0.71
HPNP_40	0.4	0.4	0.4	0.31	0.7	0.6	0.4	0.4	0.4	0.31	0.6	0.46	0.4	0.4	0.4	0.36	0.66	0.62	0.4	0.4	0.4	0.34	0.63	0.55
HPMP_40	0.42	0.51	0.49	0.33	0.76	0.65	0.42	0.49	0.46	0.34	0.67	0.5	0.43	0.5	0.49	0.39	0.72	0.69	0.43	0.49	0.48	0.37	0.69	0.61
HPHP_40	0.46	0.66	0.61	0.36	0.84	0.71	0.46	0.61	0.54	0.37	0.75	0.56	0.48	0.64	0.62	0.44	0.8	0.76	0.48	0.63	0.59	0.42	0.77	0.68
MPNP_30	0.4	0.4	0.4	0.35	0.65	0.6	0.4	0.4	0.4	0.35	0.56	0.49	0.4	0.4	0.4	0.38	0.63	0.62	0.4	0.4	0.4	0.37	0.59	0.57
MPMP_30	0.41	0.47	0.46	0.36	0.7	0.64	0.41	0.46	0.44	0.36	0.6	0.53	0.42	0.47	0.47	0.39	0.68	0.67	0.42	0.46	0.46	0.38	0.64	0.61
MPHP_30	0.43	0.59	0.56	0.37	0.77	0.7	0.43	0.54	0.5	0.38	0.66	0.57	0.44	0.57	0.57	0.42	0.75	0.74	0.44	0.55	0.54	0.41	0.7	0.67
LPNP_20	0.4	0.4	0.4	0.4	0.6	0.6	0.4	0.4	0.4	0.4	0.53	0.53	NaN	0.4	0.4	NaN	0.61	0.61	0.4	0.4	0.4	0.4	0.58	0.58
LPMP_20	0.4	0.44	0.44	0.4	0.64	0.63	0.4	0.43	0.43	0.4	0.55	0.55	NaN	0.44	0.44	NaN	0.65	0.65	0.4	0.44	0.44	0.4	0.61	0.61
LPHP_20	0.4	0.51	0.5	0.4	0.68	0.68	0.4	0.47	0.47	0.4	0.59	0.59	NaN	0.51	0.51	NaN	0.7	0.7	0.4	0.49	0.49	0.4	0.66	0.66

TABLE 3 True response rates above and below a biomarker cutoff for the two treatment groups, for each distribution of the biomarker and for the following scenarios: Non-Predictive Non-Prognostic scenario without treatment effect (NPNPNT), Non-Predictive Non-Prognostic scenario with treatment effect (NPNPT), Non-Predictive Prognostic scenario with treatment effect (NPPT), Non-Predictive Prognostic scenario with treatment effect (NPPT), High Predictive No Prognostic scenario (HPNP_50), High Predictive No Prognostic scenario (HPNP_40), Medium Predictive No Prognostic scenario (MPNP_30), Low Predictive No Prognostic scenario (LPNP_20).

2.1 | Calibration of STEPP method

Various values of $r_1 \in \{5, 15, 25, 35, 45\}$ (maximum number of patients in common in each biomarker-defined subgroup) and $r_2 \in \{10, 20, 30, 40, 50\}$ (number of patients in each biomarker-defined subgroup) have been explored under the high predictive non-prognostic scenario (HPNP_50) and when the distribution of the biomarker is $X \sim U(0, 100)$ as this was found to be the configurations (scenario and distribution of the biomarker) that was leading to the highest power compared to all other settings. The obtained results are provided in Table 4. It can be seen that for the specific analysed scenario, the choice of $r_1 = 15$ and $r_2 = 40$ lead to the highest probability to declare predictiveness compared to the other values. Thus, these two parameters will be used in the simulation study described in the next section.

r_1	r_2	STEPP method - α_{STEPP} = 0.1
5	10	0.41
5	20	0.48
5	30	0.50
5	40	0.54
5	50	0.37
15	20	0.45
15	30	0.54
15	40	0.58
15	50	0.47
25	30	0.44
25	40	0.54
25	50	0.54
35	40	0.48
35	50	0.49
45	50	0.41

TABLE 4 Probability to declare predictiveness under the STEPP method (described in Section 4.4) under the HPNP1_50 scenario, with a uniform distribution of the biomarker and α_{STEPP} = 0.1

3 | SENSITIVITY ANALYSES

3.1 | Results with data generated using a step function

Additional explorations have been conducted generating data which follow a step function relationship between ORR and biomarker X, that is:

$$p_k = \begin{cases} p_{ka} & \text{if } X > c \\ p_{kb} & \text{if } X \leq c \end{cases} \qquad \text{ORR} \sim Binom(N, p)$$

where p_{ka}, p_{kb} are the response rates above and below the cut-off c for arm $k \in \{0, 1\}$ respectively and N is the sample size. These response rates are summarised in Table 5 and examples of scenarios are provided in Figure 1.

Figure 2 summarises the results for all methods, all null scenarios and all the considered distributions of the biomarker. Overall, similar patterns between the methods to those observed in the previous section can be observed here. It can be observed that under the null scenarios, the type I error rate is controlled at level 15% for all methods and biomarker scenarios. The only exception, is when the type I error rate is slightly inflated for the interaction test method under the NPPT scenario when the biomarker is Skewed gamma.

Figure 3 provides the results for all methods and the non-null scenarios considered in the study. In general, the results show a much lower (below around 70% when the sample size is unbalanced) power for all methods, scenarios and distributions of the biomarker compared to the results where the ORR-biomarker relationship is logistic. Moreover, under the scenarios where the predictive effect is medium or low, the probability of detecting the predictive value is closer to the nominal alpha level of 15%. The novel approach here shows similar results to the DeLong test for all considered cases.

3.2 | Results with a balanced sample size

Figures 4 and 5 summarise the results for all methods and all the considered distributions of the biomarker when the sample size is balanced between the experimental and the control arms, that is 40 patients in each treatment group, for all null and non-null scenarios, respectively, when the data are generated considering a logistic distribution between the ORR and the biomarker values (as described in Section 5.1).

Figures 6 and 7 summarise the results for all methods and all the considered distributions of the biomarker when the sample size is balanced between the experimental and the control arms, that is 40 patients in each treatment group, for all null and non-null scenarios, respectively, when the data are generated considering a step function between the ORR and the biomarker values (as described above).

Overall, similar patterns of the results to those observed in Section 5.3 can be observed here. Under the null scenarios, the type I error rate is controlled at level 15% for all methods (except for the interaction test with binary outcome method where there is an inflation up to 7% and slightly for the IT and AKSA methods in some scenarios) and distributions of the biomarker.

The novel approach leads in general to similar or slightly higher power compared to the DeLong test and to the standard interaction test (up to around 15%). All other methods lead to a lower power to detect a predictive value of the biomarker. In general, the balanced sample size leads to higher power compared to the 40:20 setting (for example when the biomarker is uniform and the sample size is 40:40, the power for the novel approach ranges from around 40% up to 92% for all non-null scenarios compared to the other distributions of the biomarker).

Figures 8 and 9 summarise the results for all methods and all the considered distributions of the biomarker when the sample size is balanced between the experimental and the control arms, that is 100 patients in each treatment group, for all null and non-null scenarios, respectively, when the data are generated considering a logistic distribution between the ORR and the biomarker values (as described in Section 5.1).

Figures 10 and 11 summarise the results for all methods and all the considered distributions of the biomarker when the sample size is balanced between the experimental and the control arms, that is 100 patients in each treatment group, for all null and non-null scenarios, respectively, when the data are generated considering a step function between the ORR and the biomarker values (as described above).

Overall, similar patterns of the results to those observed before can be observed here.

3.3 | Results with data generated using a step function

	Cutoff biomarker (c)	Placebo			Experimental		
		p_{0b}	p_{0a}	Overall Rate	p_{1b}	p_{1a}	Overall Rate
NPNPT	NA	NA	NA	0.4	NA	NA	0.6
NPPT	17	0.19	0.47	0.42	0.35	0.65	0.6
NPNPNT	NA	NA	NA	0.4	NA	NA	0.4
NPPNT	17	0.19	0.47	0.42	0.19	0.47	0.42
HPNP_50	30	0.4	0.4	0.4	0.26	0.75	0.6
HPMP_50	30	0.44	0.56	0.52	0.29	0.83	0.67
HPHP_50	30	0.49	0.76	0.68	0.34	0.9	0.73
HPNP_40	26	0.4	0.4	0.4	0.31	0.7	0.6
HPMP_40	26	0.42	0.51	0.49	0.33	0.76	0.65
HPHP_40	26	0.46	0.66	0.61	0.36	0.84	0.71
MPNP_30	17	0.4	0.4	0.4	0.35	0.65	0.6
MPMP_30	17	0.41	0.47	0.46	0.36	0.7	0.64
MPHP_30	17	0.43	0.59	0.56	0.37	0.77	0.7
LPNP_20	1	0.4	0.4	0.4	0.4	0.6	0.6
LPMP_20	1	0.4	0.44	0.44	0.4	0.64	0.63
LPHP_20	1	0.4	0.51	0.5	0.4	0.68	0.68

TABLE 5 True response rates above and below a biomarker cutoff for the two treatment groups and for all considered scenarios.

Examples of the considered scenarios are represented in Figures 1.

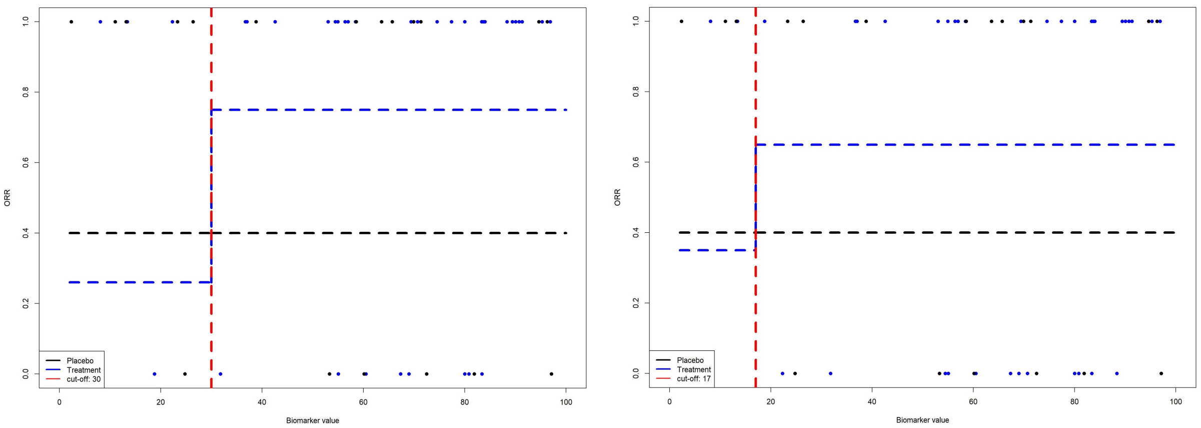

FIGURE 1 True alternative scenarios for step function: HPNP-50 High Predictive No Prognostic scenario with 50% predictive effect (left); MPNP-30 Moderate Predictive No Prognostic scenario with 30% predictive effect (right).

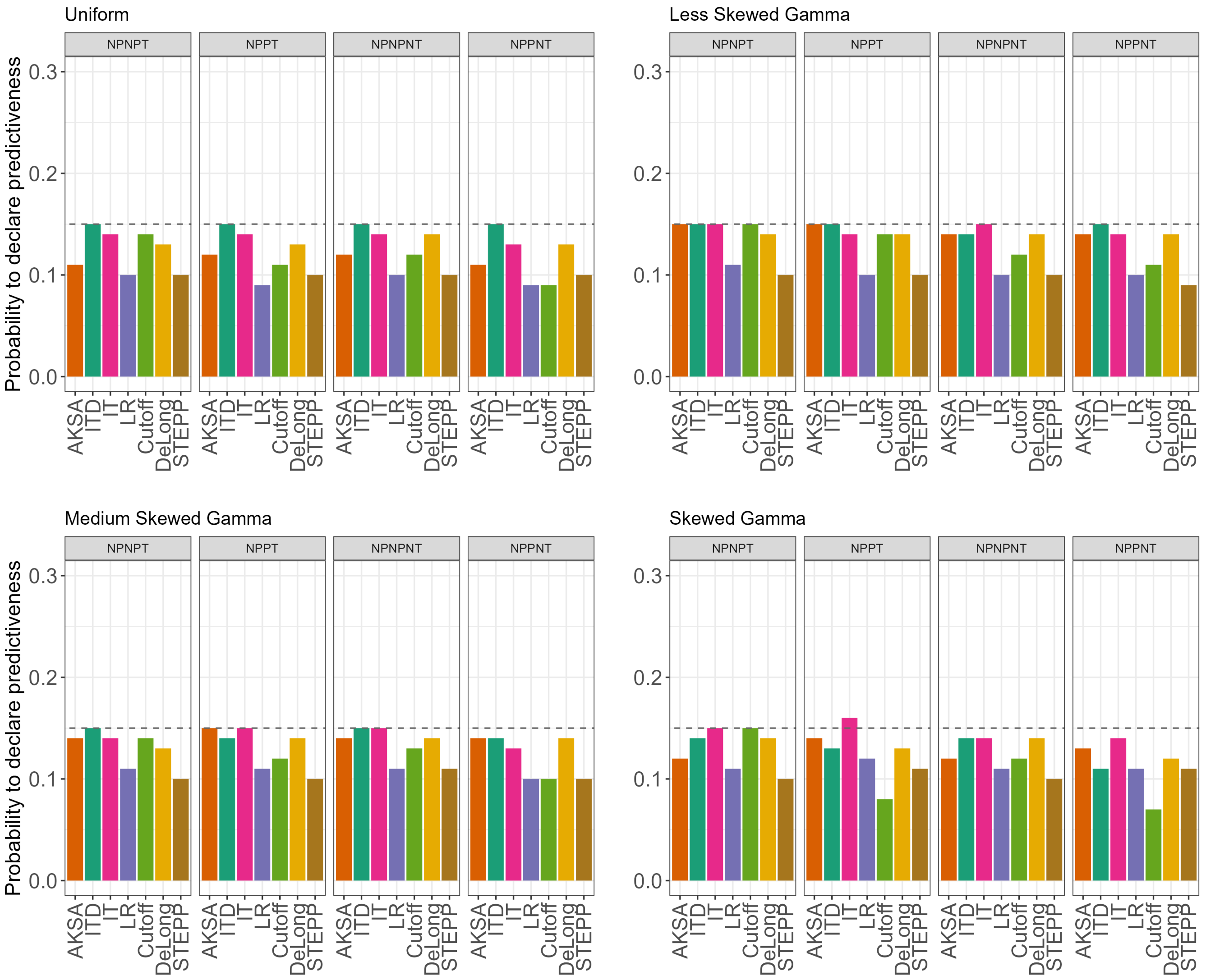

FIGURE 2 Results for all null scenarios and for all approaches: interaction test (IT), interaction test with a dichotomous bmk (ITD), likelihood ratio test (LR), STEPP approach (STEPP), probability to find a cutoff (Cutoff), DeLong test (DeLong), difference between two curves (AKSA) for different distributions of the biomarker and when the sample size is 40:20 patients (experimental:control). Data are generated using a step function.

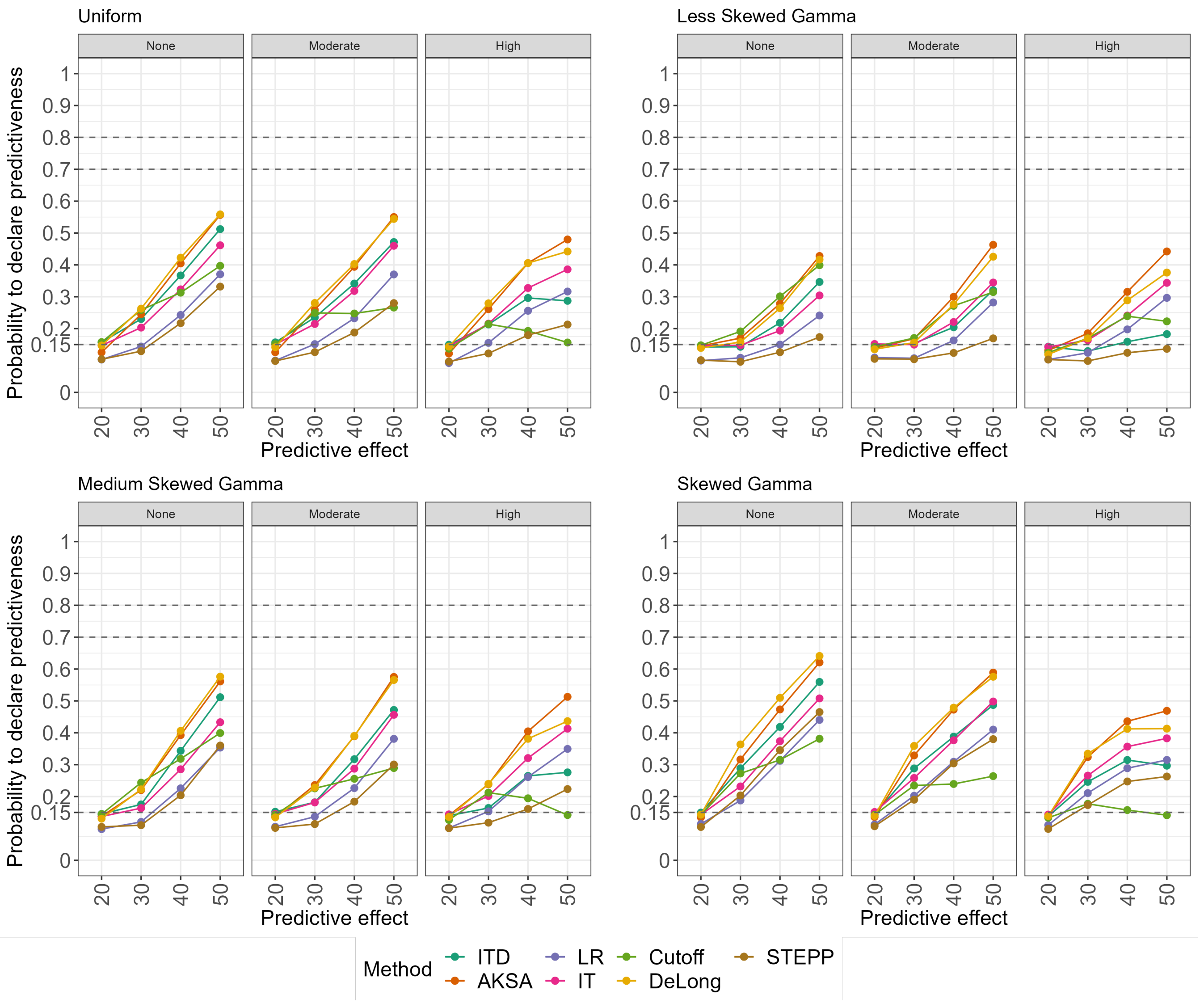

FIGURE 3 Results for all non-null scenarios and for all approaches: interaction test (IT), interaction test with a dichotomous bmk (ITD), likelihood ratio test (LR), STEPP approach (STEPP), probability to find a cutoff (Cutoff), DeLong test (DeLong), difference between two curves (AKSA) for different distributions of the biomarker and whenthe sample size is 40:20 patients (experimental:control). Data are generated using a step function. Columns in the graphs represent the prognostic effect (N = null, M = moderate and H=high). On the x-axis instead the predictive effect is reported (20%, 30%, 40% or 50%).

3.4 | Results with a balanced sample size

3.4.1 | Total number of patients equal to 80

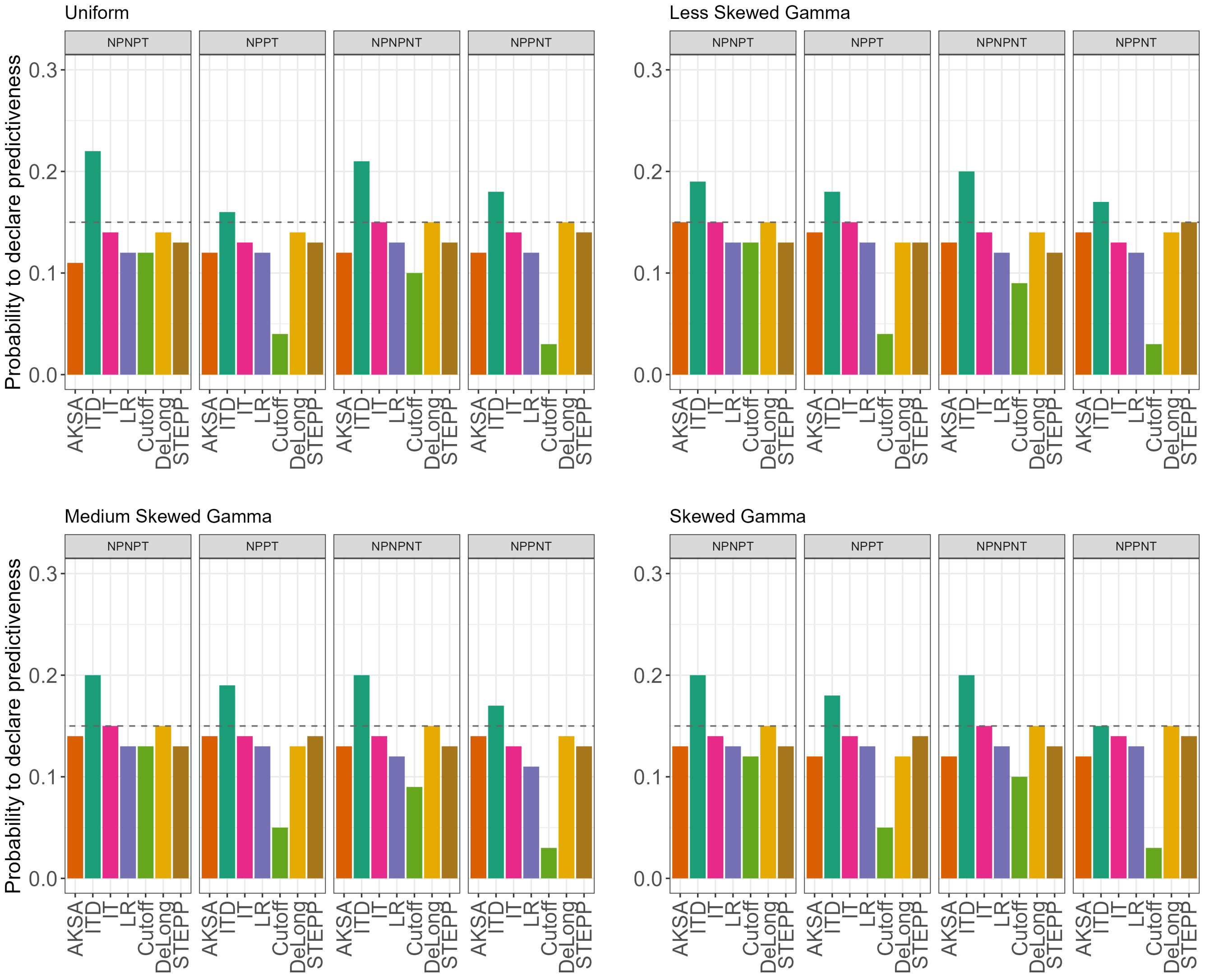

FIGURE 4 Results for all null scenarios and for all approaches: interaction test (IT), interaction test with a dichotomous bmk (ITD), likelihood ratio test (LR), STEPP approach (STEPP), probability to find a cutoff (Cutoff), DeLong test (DeLong), difference between two curves (AKSA) for different distributions of the biomarker and when the sample size is 40:40 patients (experimental:control). Biomarker-response data are generated following a logistic function.

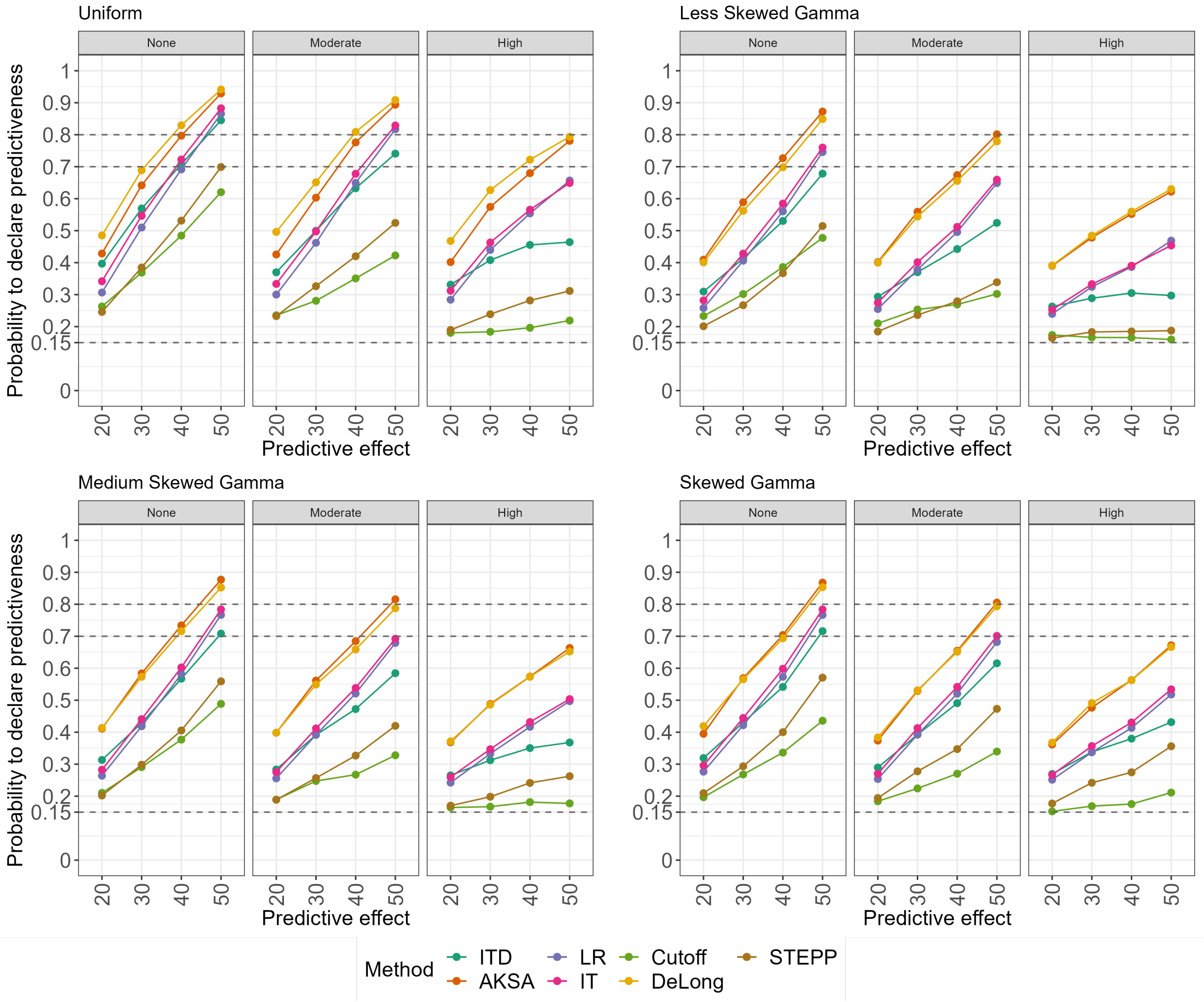

FIGURE 5 Results for all non-null scenarios and for all approaches: interaction test (IT), interaction test with a dichotomous bmk (ITD), likelihood ratio test (LR), STEPP approach (STEPP), probability to find a cutoff (Cutoff), DeLong test (DeLong), difference between two curves (AKSA) for different distributions of the biomarker and when the sample size is 40:40 patients (experimental:control). Columns in the graphs represent the prognostic effect (N = null, M = moderate and H=high). On the x-axis instead the predictive effect is reported (20%, 30%, 40% or 50%). Biomarker-response data are generated following a logistic function.

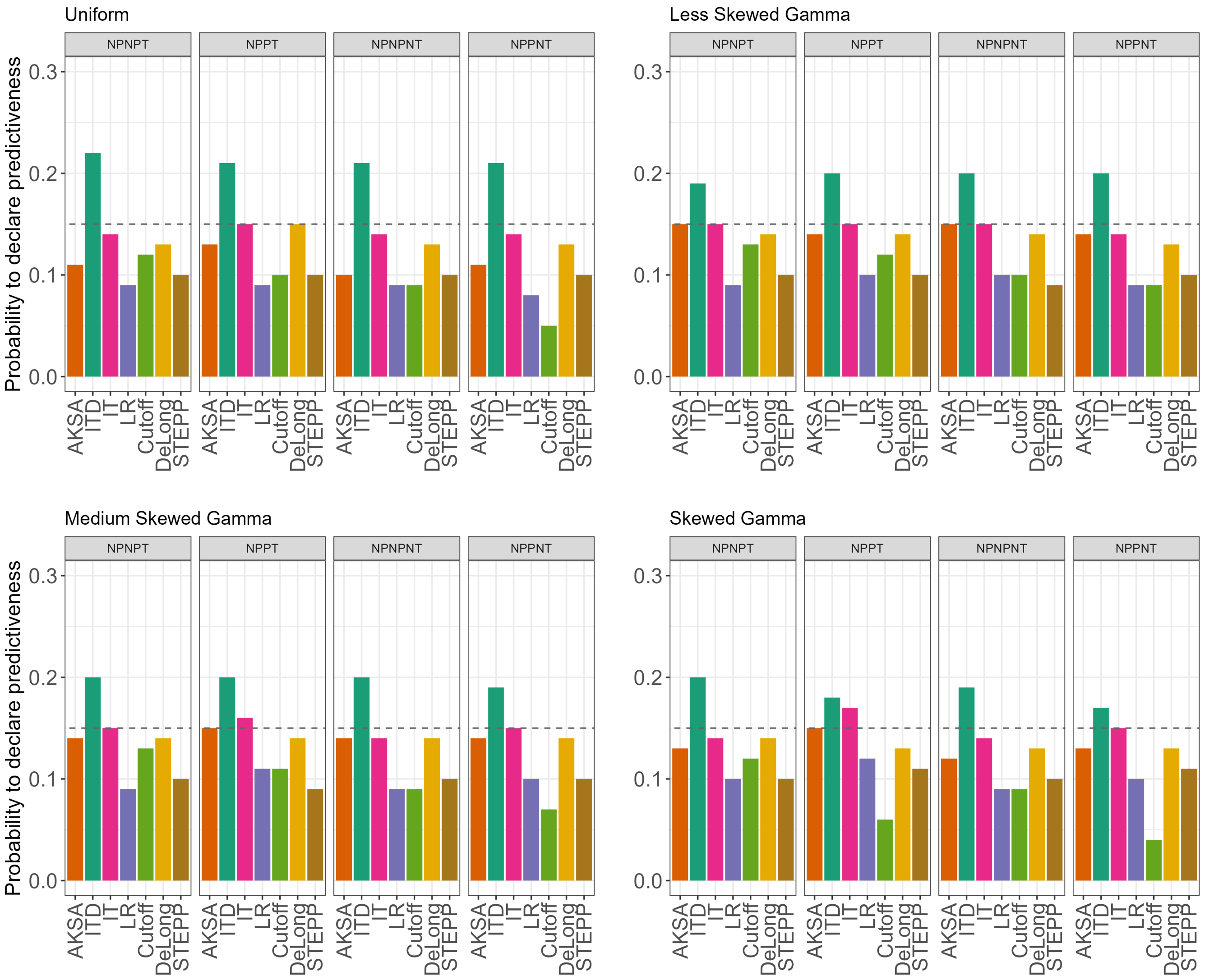

FIGURE 6 Results for all null scenarios and for all approaches: interaction test (IT), interaction test with a dichotomous bmk (ITD), likelihood ratio test (LR), STEPP approach (STEPP), probability to find a cutoff (Cutoff), DeLong test (DeLong), difference between two curves (AKSA) for different distributions of the biomarker and when the sample size is 40:40 patients (experimental:control). Data are generated using a step function.

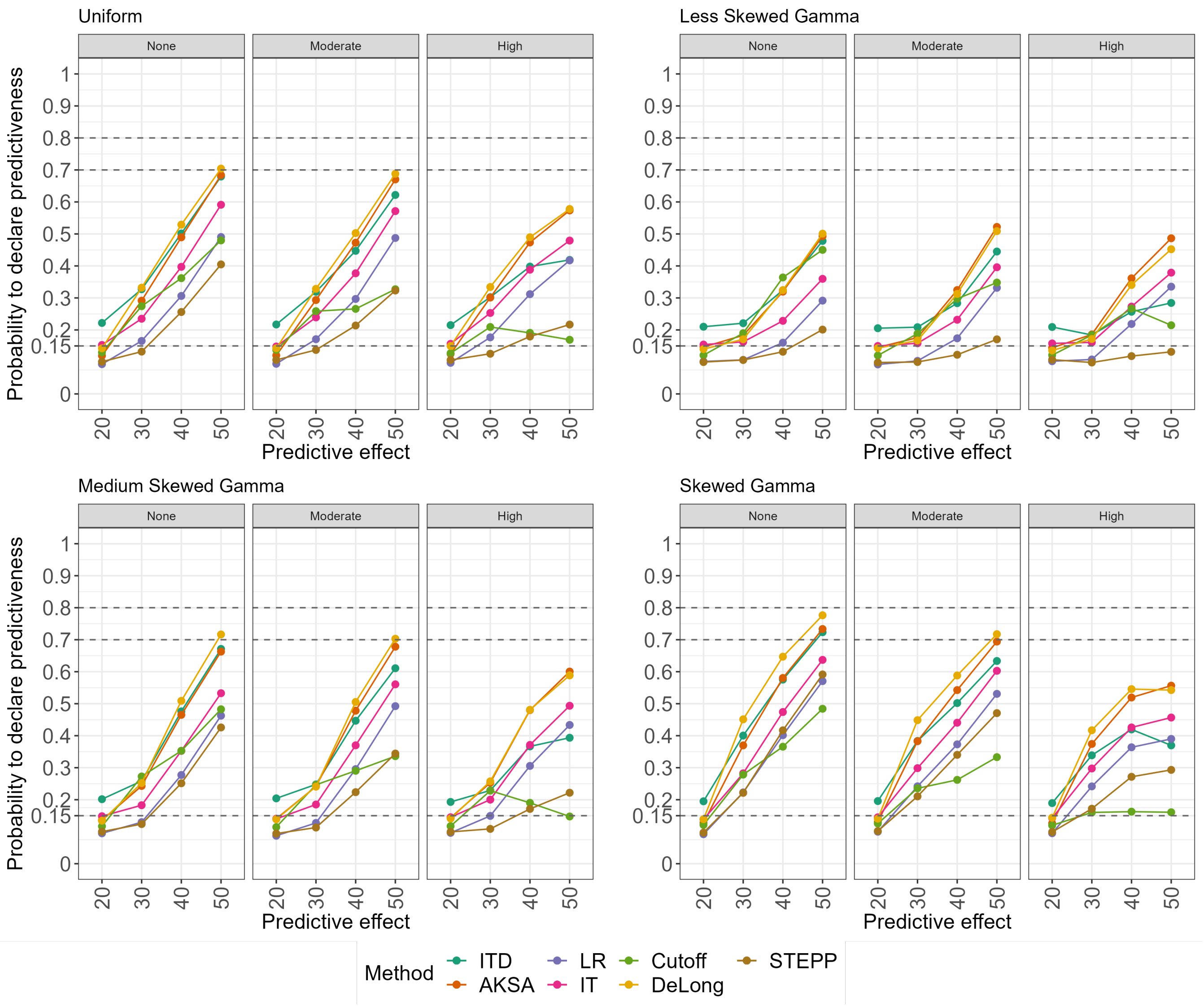

FIGURE 7 Results for all non-null scenarios and for all approaches: interaction test (IT), interaction test with a dichotomous bmk (ITD), likelihood ratio test (LR), STEPP approach (STEPP), probability to find a cutoff (Cutoff), DeLong test (DeLong), difference between two curves (AKSA) for different distributions of the biomarker and when the sample size is 40:40 patients (experimental:control). Data are generated using a step function. Columns in the graphs represent the prognostic effect (N = null, M = moderate and H=high). On the x-axis instead, the predictive effect is reported (20%, 30%, 40% or 50%).

3.4.2 | Total number of patients equal to 200

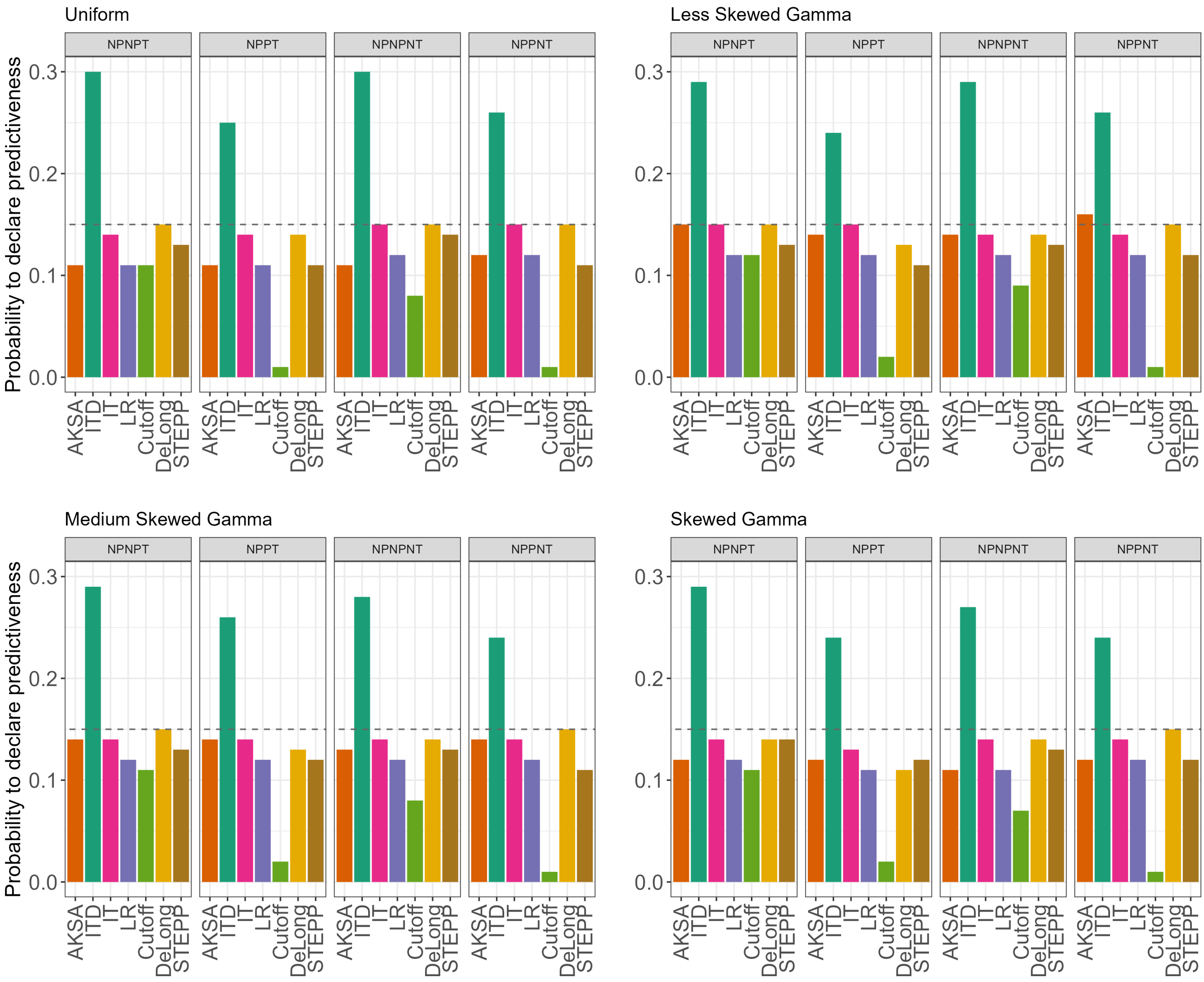

FIGURE 8 Results for all null scenarios and for all approaches: interaction test (IT), interaction test with a dichotomous bmk (ITD), likelihood ratio test (LR), STEPP approach (STEPP), probability to find a cutoff (Cutoff), DeLong test (DeLong), difference between two curves (AKSA) for different distributions of the biomarker and when the sample size is 100:100 patients (experimental:control). Biomarker-response data are generated following a logistic distribution.

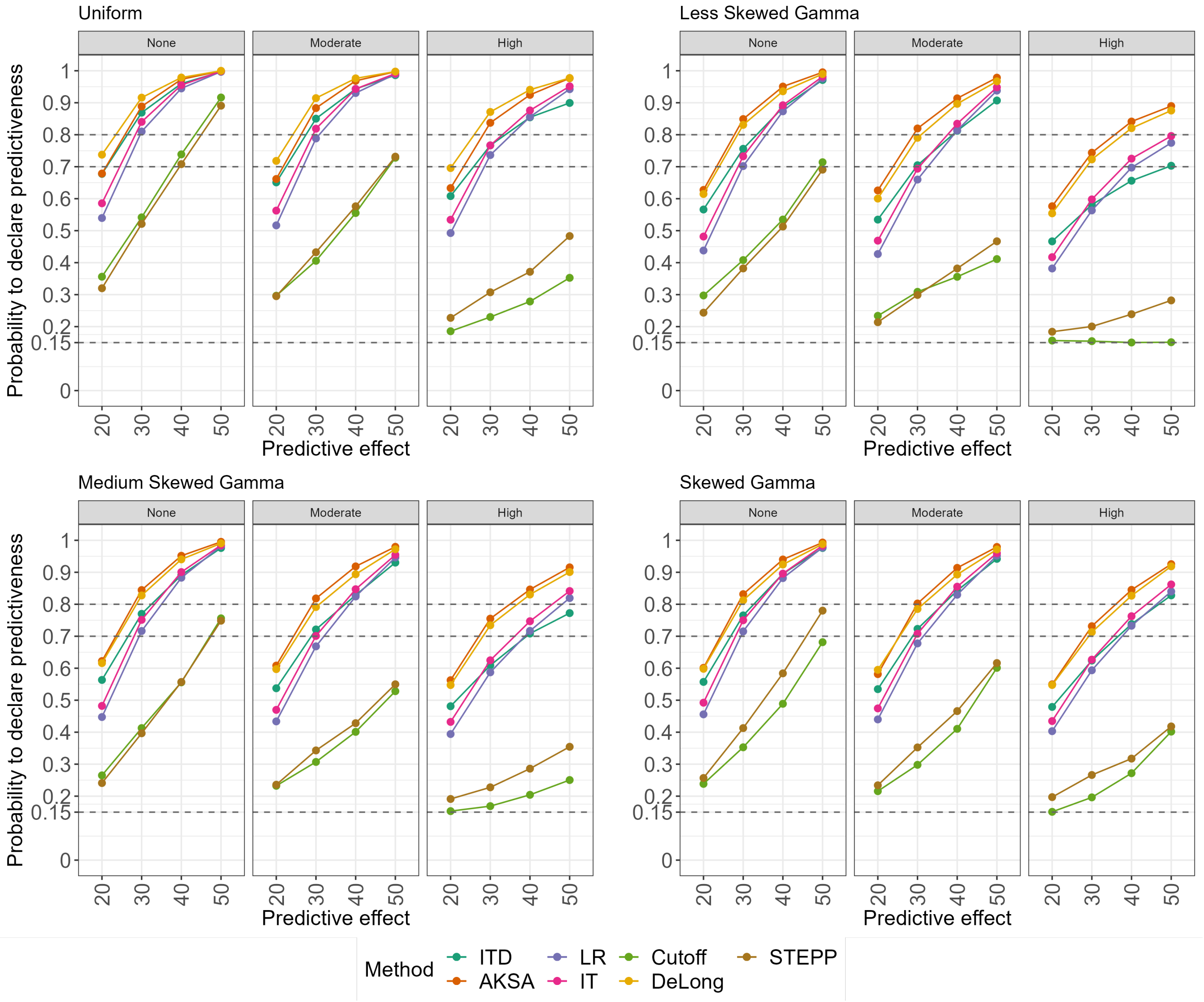

FIGURE 9 Results for all non-null scenarios and for all approaches: interaction test (IT), interaction test with a dichotomous bmk (ITD), likelihood ratio test (LR), STEPP approach (STEPP), probability to find a cutoff (Cutoff), DeLong test (DeLong), difference between two curves (AKSA) for different distributions of the biomarker and when the sample size is 100:100 patients (experimental:control). Columns in the graphs represent the prognostic effect (N = null, M = moderate and H=high). On the x-axis instead the predictive effect is reported (20%, 30%, 40% or 50%). Biomarker-response data are generated following a logistic distribution.

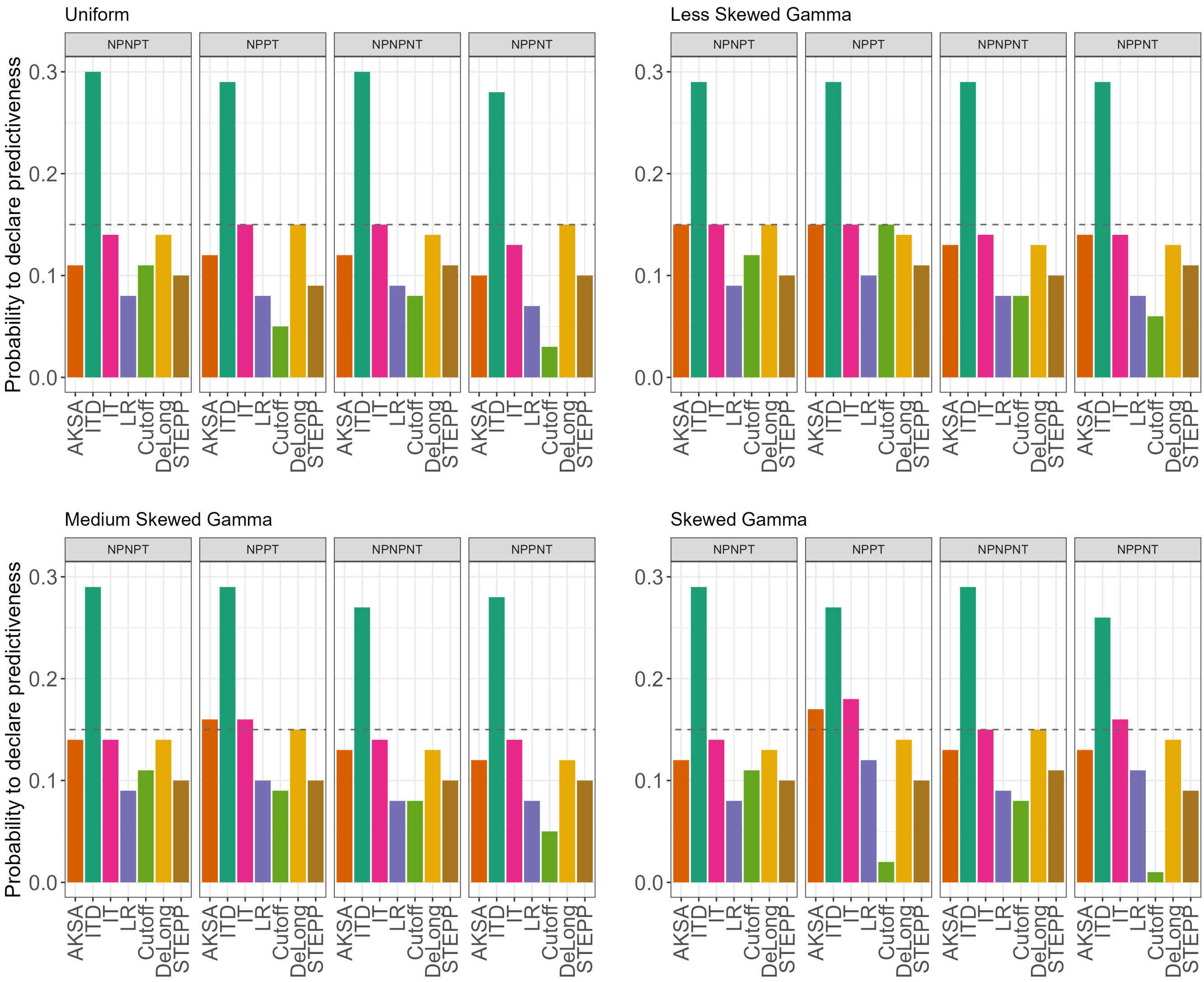

FIGURE 10 Results for all null scenarios and for all approaches: interaction test (IT), interaction test with a dichotomous bmk (ITD), likelihood ratio test (LR), STEPP approach (STEPP), probability to find a cutoff (Cutoff), DeLong test (DeLong), difference between two curves (AKSA) for different distributions of the biomarker and when the sample size is 100:100 patients (experimental:control). Data are generated using a step function.

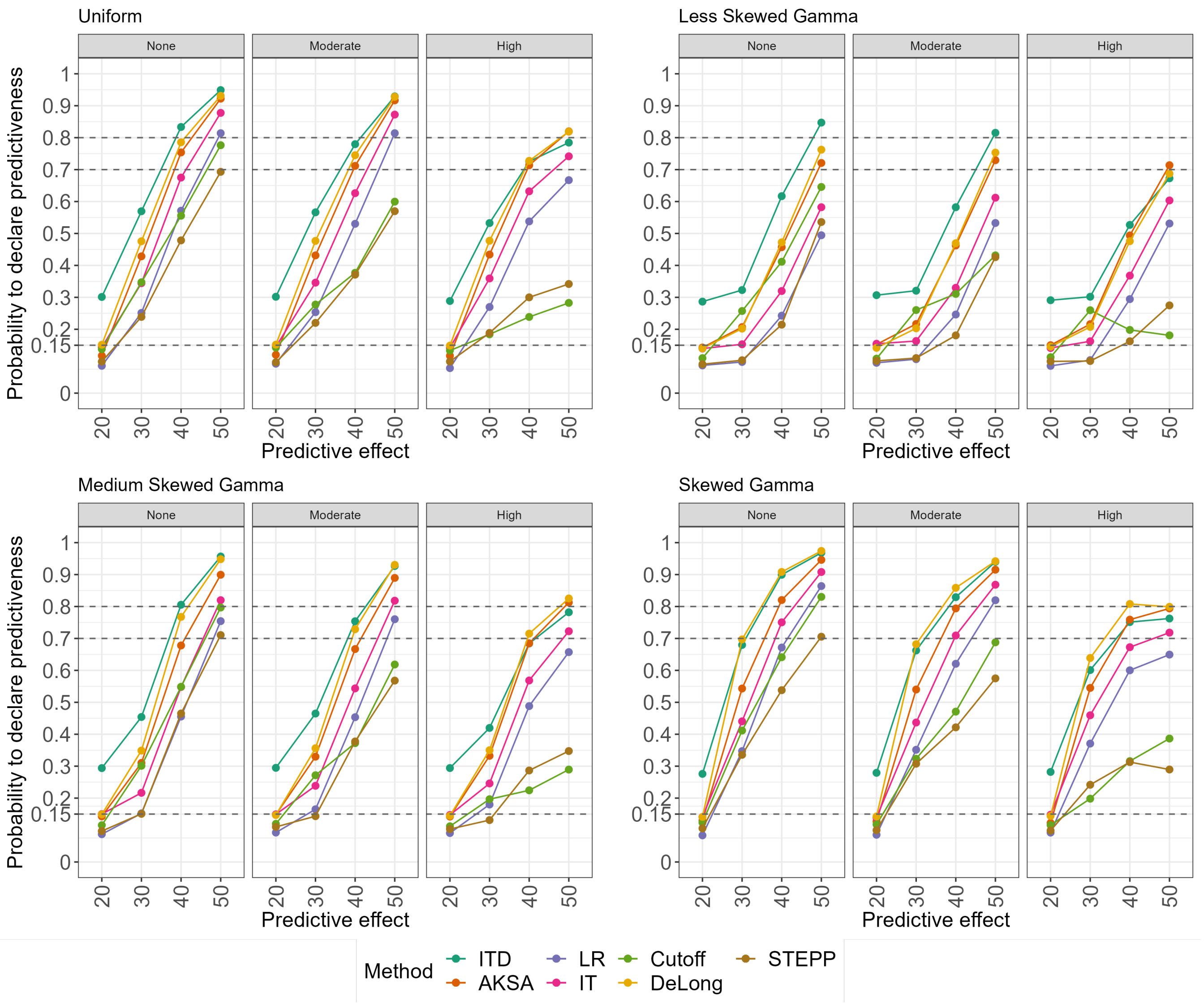

FIGURE 11 Results for all non-null scenarios and for all approaches: interaction test (IT), interaction test with a dichotomous bmk (ITD), likelihood ratio test (LR), STEPP approach (STEPP), probability to find a cutoff (Cutoff), DeLong test (DeLong), difference between two curves (AKSA) for different distributions of the biomarker and when the sample size is 100:100 patients (experimental:control). Data are generated using a step function. Columns in the graphs represent the prognostic effect (N = null, M = moderate and H=high). On the x-axis instead the predictive effect is reported (20%, 30%, 40% or 50%).

3.5 | Total number of patients equal to 30

For this specific setting the pre-specified thresholds are set as: α_{IT} = 0.17 (Interaction test), α_{ITD} = 0.09 (Interaction test dichotomized biomarker), α_{LR} = 0.06 (Likelihood ratio test), α_{STEPP} = 0.13 (STEPP approach), α_{cutoff} = 0.9985 (Probability to find a cutoff), α_{DeLong} = 0.145 (DeLong test), α_{AKSA} = 0.735 (Difference between two curves).

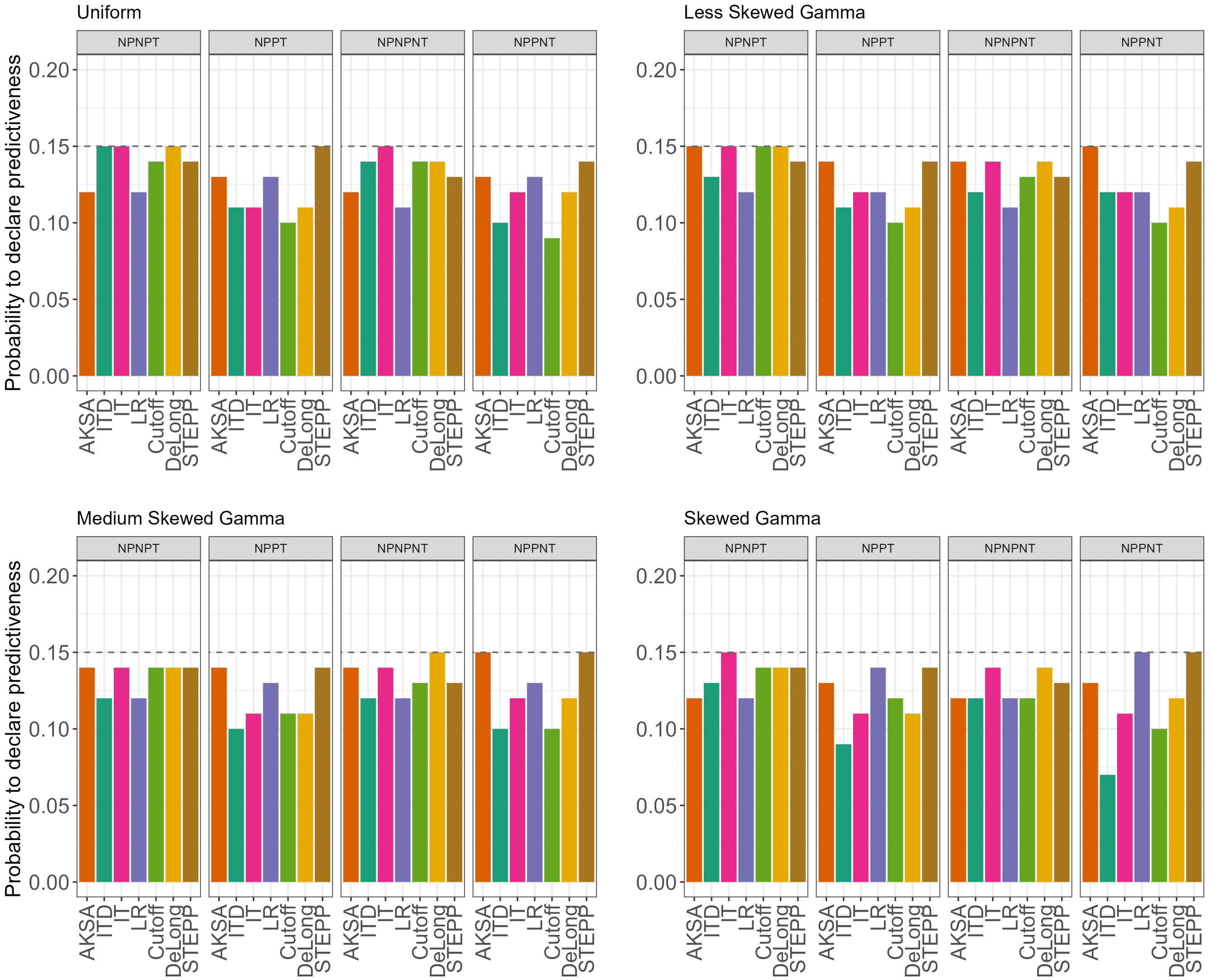

FIGURE 12 Results for all null scenarios and for all approaches: interaction test (IT), interaction test with a dichotomous bmk (ITD), likelihood ratio test (LR), STEPP approach (STEPP), probability to find a cutoff (Cutoff), DeLong test (DeLong), difference between two curves (AKSA) for different distributions of the biomarker and when the sample size is 20:10 patients (experimental:control). Biomarker-response data are generated following a logistic distribution.

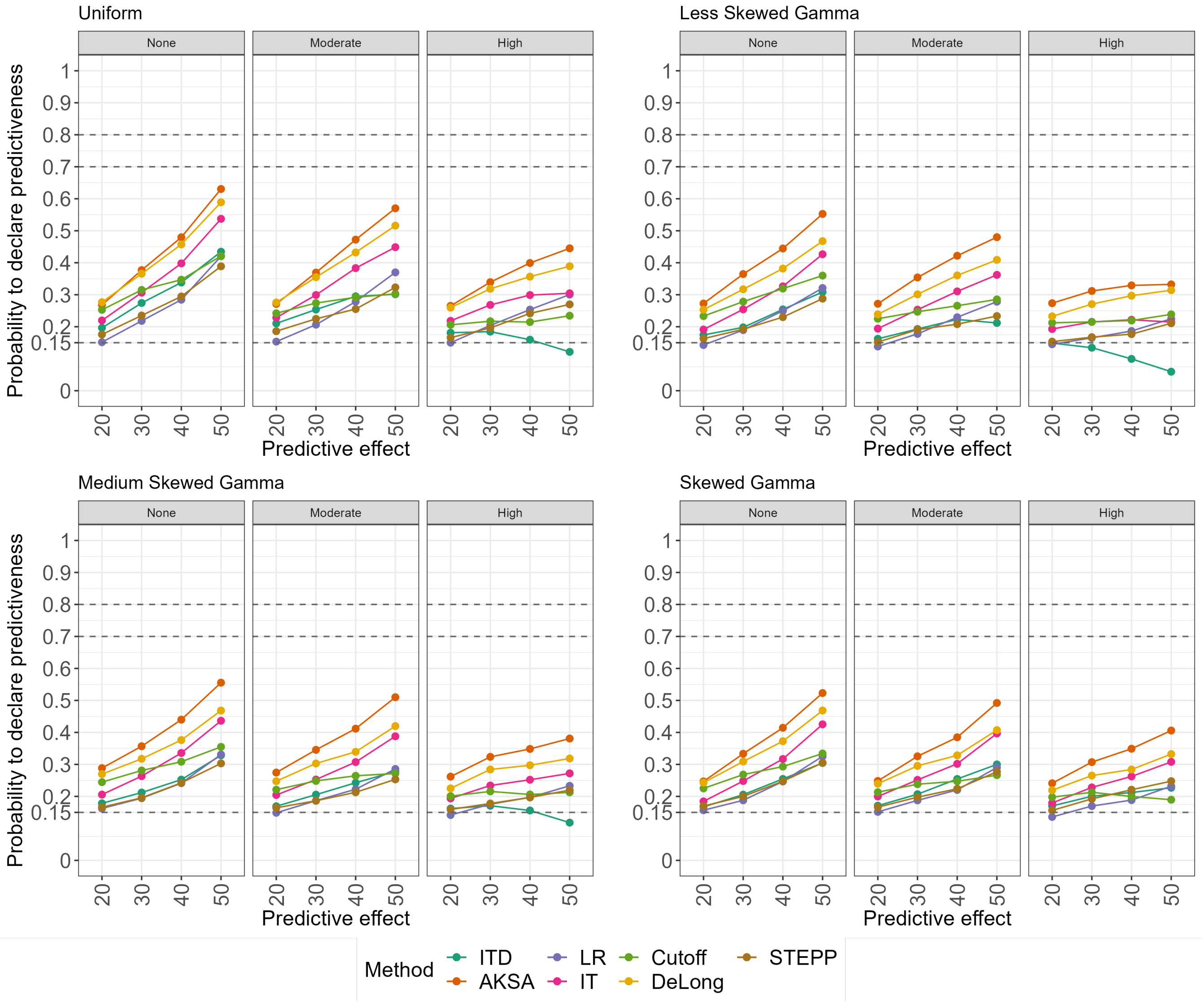

FIGURE 13 Results for all non-null scenarios and for all approaches: interaction test (IT), interaction test with a dichotomous bmk (ITD), likelihood ratio test (LR), STEPP approach (STEPP), probability to find a cutoff (Cutoff), DeLong test (DeLong), difference between two curves (AKSA) for different distributions of the biomarker and when the sample size is 20:10 patients (experimental:control). Columns in the graphs represent the prognostic effect (N = null, M = moderate and H=high). On the x-axis instead the predictive effect is reported (20%, 30%, 40% or 50%). Biomarker-response data are generated following a logistic distribution.

4 | ADDITIONAL SCENARIOS

Coefficients (term)	HPNP2_50	HPNP2_40	MPNP2_30	LPNP2_20
b_0	-0.405	-0.405	-0.405	-0.405
b_1	-1.863	-1.36	-0.853	-0.465
b_2	0.045	0.034	0.024	0.017
b_3	0	0	0	0
True cutoff	42	40	35	28

TABLE 6 True coefficients and true cutoff values for the logistic regression model for null scenarios and the alternative scenarios without a prognostic effect.

	Placebo			Experimental		
	Rate Below cut-off	Rate Above cut-off	Overall Rate	Rate Below cut-off	Rate Above cut-off	Overall Rate
HPNP2_50	0.4	0.4	0.4	0.22	0.69	0.5
HPMP2_50	0.45	0.56	0.51	0.26	0.79	0.57
HPHP2_50	0.52	0.76	0.66	0.32	0.89	0.65
HPNP2_40	0.4	0.4	0.4	0.26	0.64	0.49
HPMP2_40	0.43	0.52	0.48	0.29	0.73	0.55
HPHP2_40	0.48	0.68	0.6	0.33	0.83	0.63
MPNP2_30	0.4	0.4	0.4	0.3	0.59	0.49
MPMP2_30	0.42	0.48	0.46	0.32	0.66	0.54
MPHP2_30	0.45	0.6	0.55	0.35	0.74	0.61
LPNP2_20	0.4	0.4	0.4	0.35	0.55	0.5
LPMP2_20	0.41	0.45	0.44	0.36	0.6	0.53
LPHP2_20	0.43	0.53	0.5	0.38	0.67	0.59

TABLE 7 True response rates above and below a biomarker cutoff for the two treatment groups and for the second set of scenarios

4.1 | Biomarker-response data are generated following a logistic function

4.1.1 | Results with a total sample size of 60 (40:20) patients

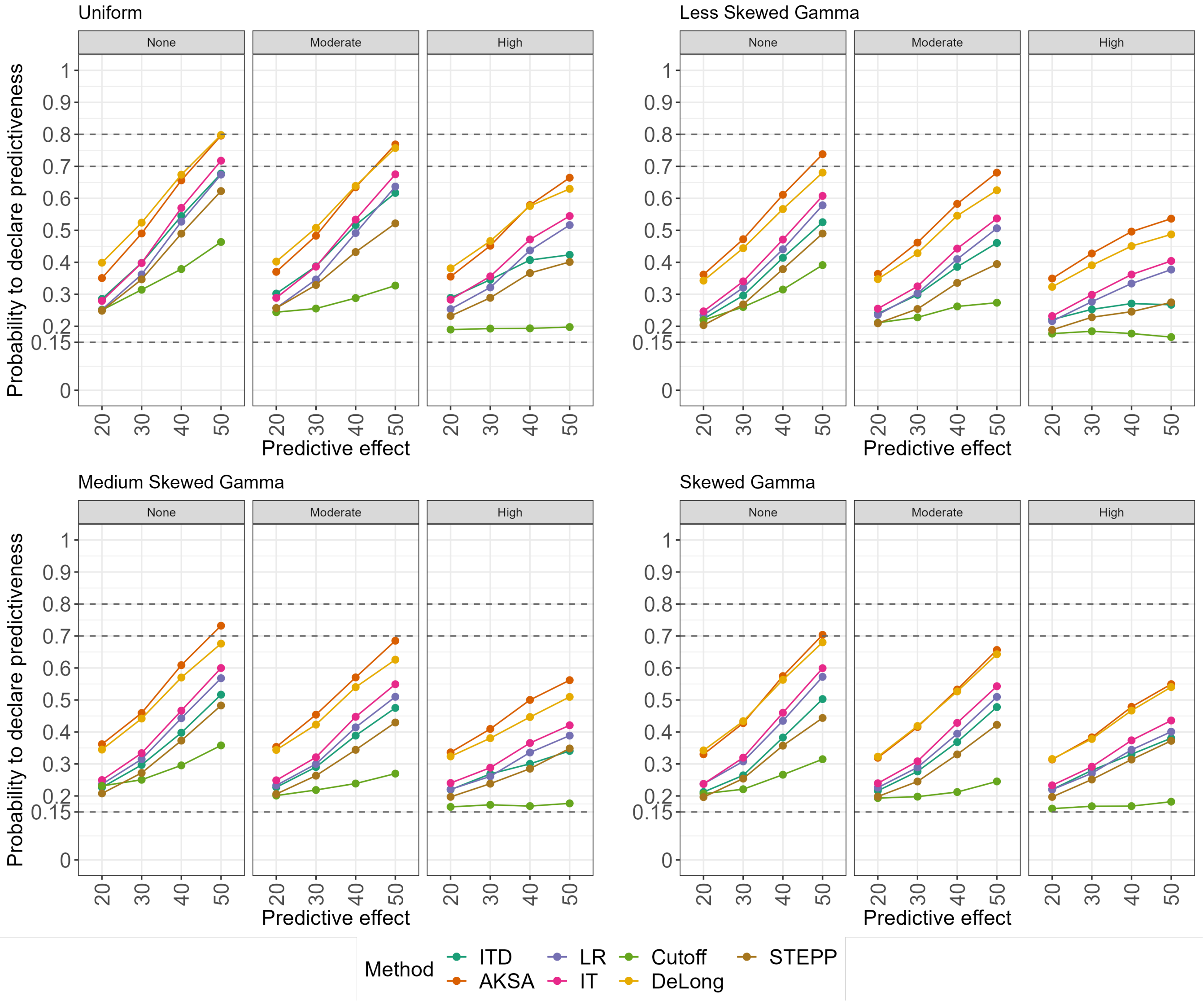

FIGURE 14 Results for all non-null scenarios and for all approaches: interaction test (IT), interaction test with a dichotomous bmk (ITD), likelihood ratio test (LR), STEPP approach (STEPP), probability to find a cutoff (Cutoff), DeLong test (DeLong), difference between two curves (AKSA) for different distributions of the biomarker and when the sample size is 40:20 patients (experimental:control). Columns in the graphs represent the prognostic effect (N = null, M = moderate and H=high). On the x-axis instead the predictive effect is reported (20%, 30%, 40% or 50%). Biomarker-response data are generated following a logistic function.

4.1.2 | Results with a total sample size of 80 (40:40) patients

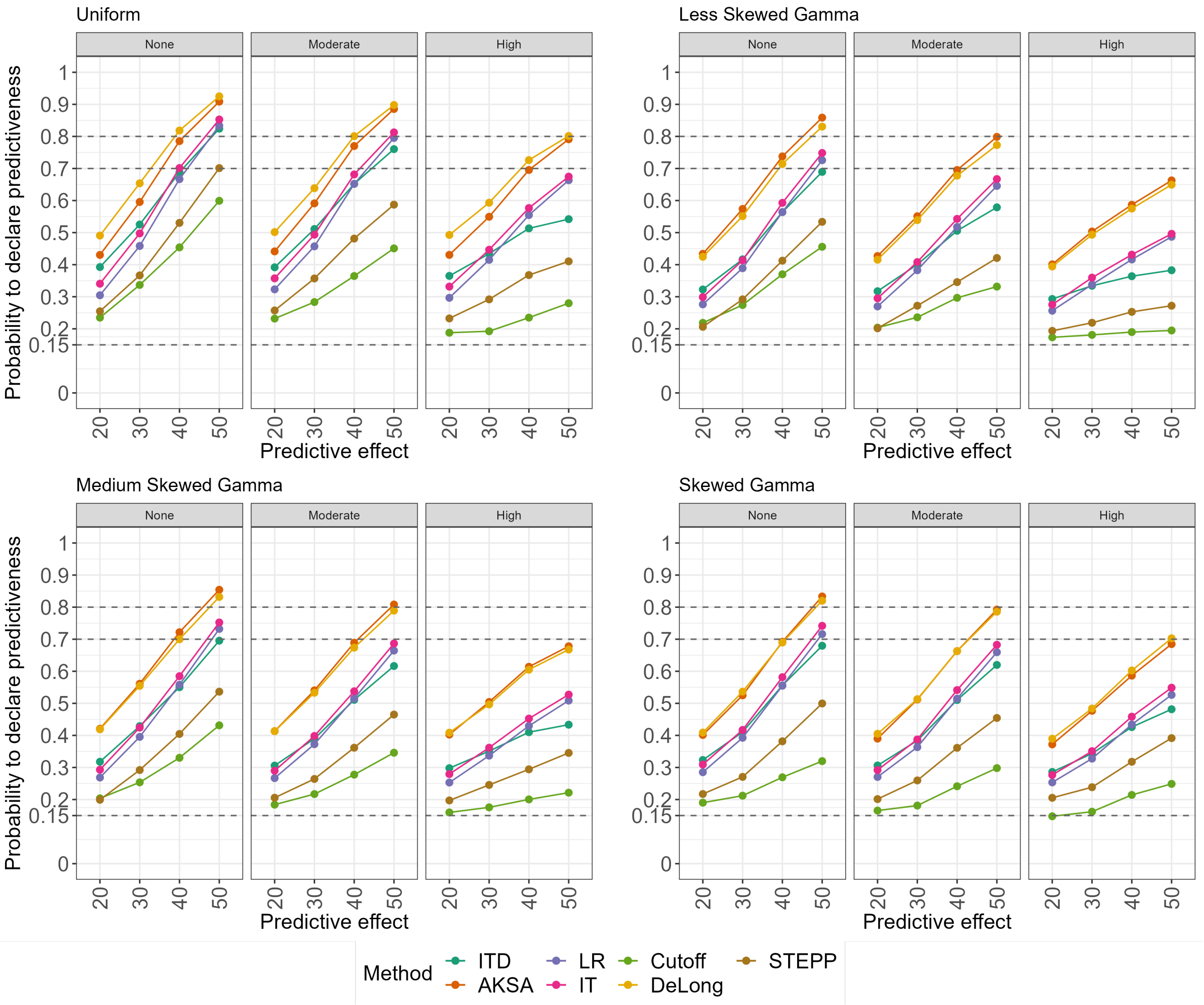

FIGURE 15 Results for all non-null scenarios and for all approaches: interaction test (IT), interaction test with a dichotomous bmk (ITD), likelihood ratio test (LR), STEPP approach (STEPP), probability to find a cutoff (Cutoff), DeLong test (DeLong), difference between two curves (AKSA) for different distributions of the biomarker and when the sample size is 40:40 patients (experimental:control). Columns in the graphs represent the prognostic effect (N = null, M = moderate and H=high). On the x-axis instead the predictive effect is reported (20%, 30%, 40% or 50%). Biomarker-response data are generated following a logistic function.

4.1.3 | Results with a total sample size of 200 (100:100) patients

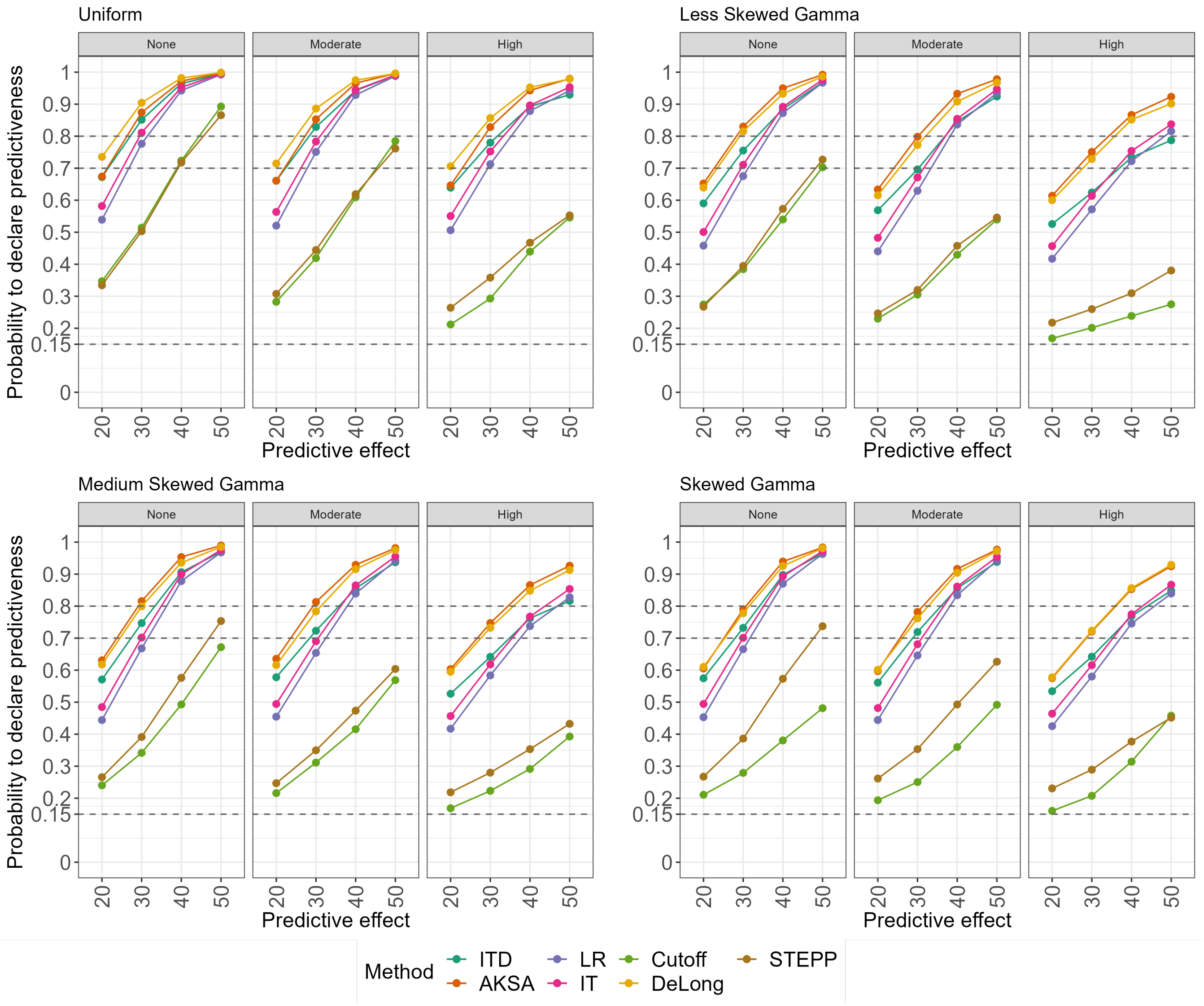

FIGURE 16 Results for all non-null scenarios and for all approaches: interaction test (IT), interaction test with a dichotomous bmk (ITD), likelihood ratio test (LR), STEPP approach (STEPP), probability to find a cutoff (Cutoff), DeLong test (DeLong), difference between two curves (AKSA) for different distributions of the biomarker and when the sample size is 100:100 patients (experimental:control). Columns in the graphs represent the prognostic effect (N = null, M = moderate and H=high). On the x-axis instead the predictive effect is reported (20%, 30%, 40% or 50%). Biomarker-response data are generated following a logistic function.

4.2 | Biomarker-response data are generated following a step function

4.2.1 | Results with a total sample size of 60 (40:20) patients

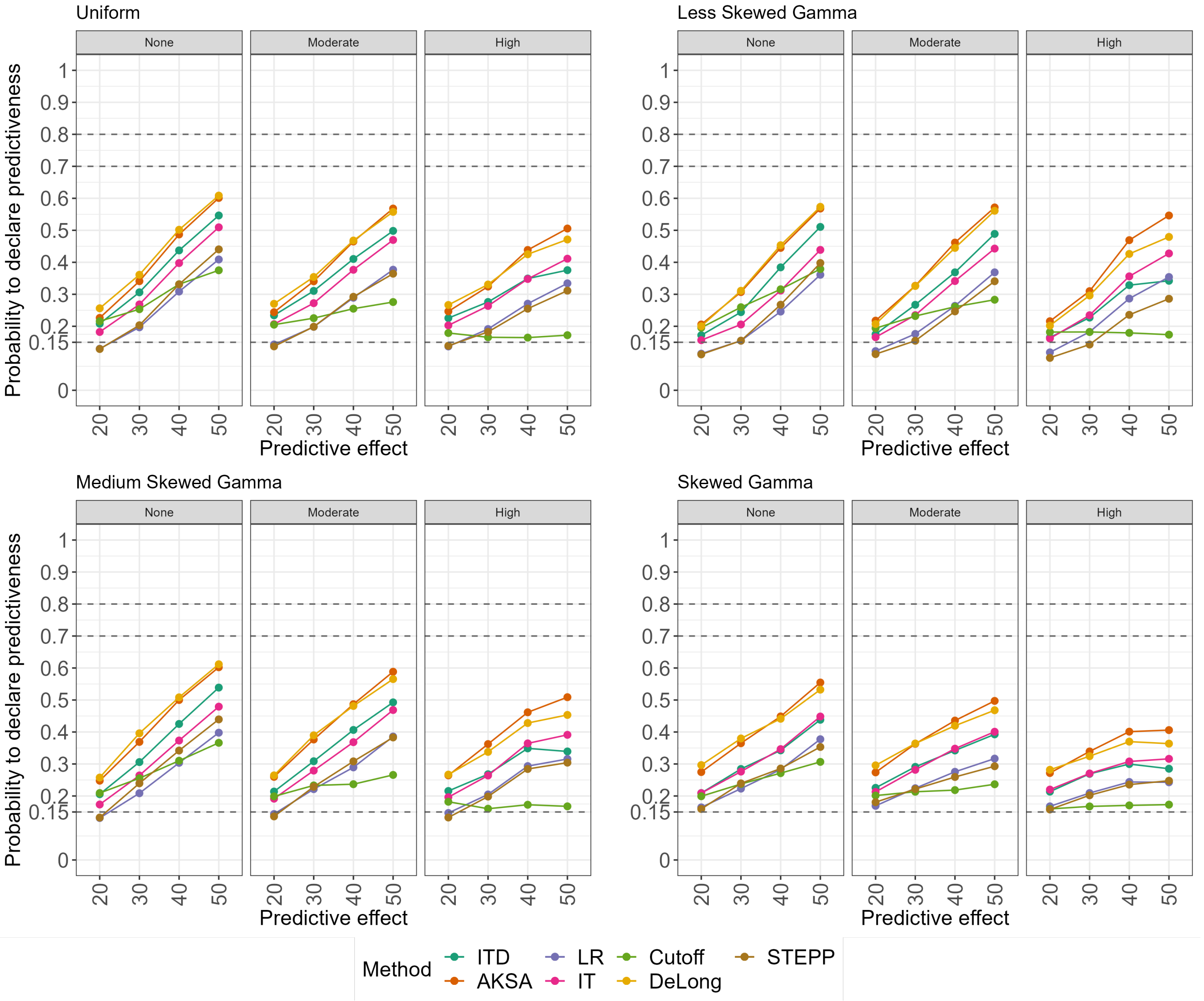

FIGURE 17 Results for all non-null scenarios and for all approaches: interaction test (IT), interaction test with a dichotomous bmk (ITD), likelihood ratio test (LR), STEPP approach (STEPP), probability to find a cutoff (Cutoff), DeLong test (DeLong), difference between two curves (AKSA) for different distributions of the biomarker and when the sample size is 40:20 patients (experimental:control). Columns in the graphs represent the prognostic effect (N = null, M = moderate and H=high). On the x-axis instead the predictive effect is reported (20%, 30%, 40% or 50%). Biomarker-response data are generated following a step function.

4.2.2 | Results with a total sample size of 80 (40:40) patients

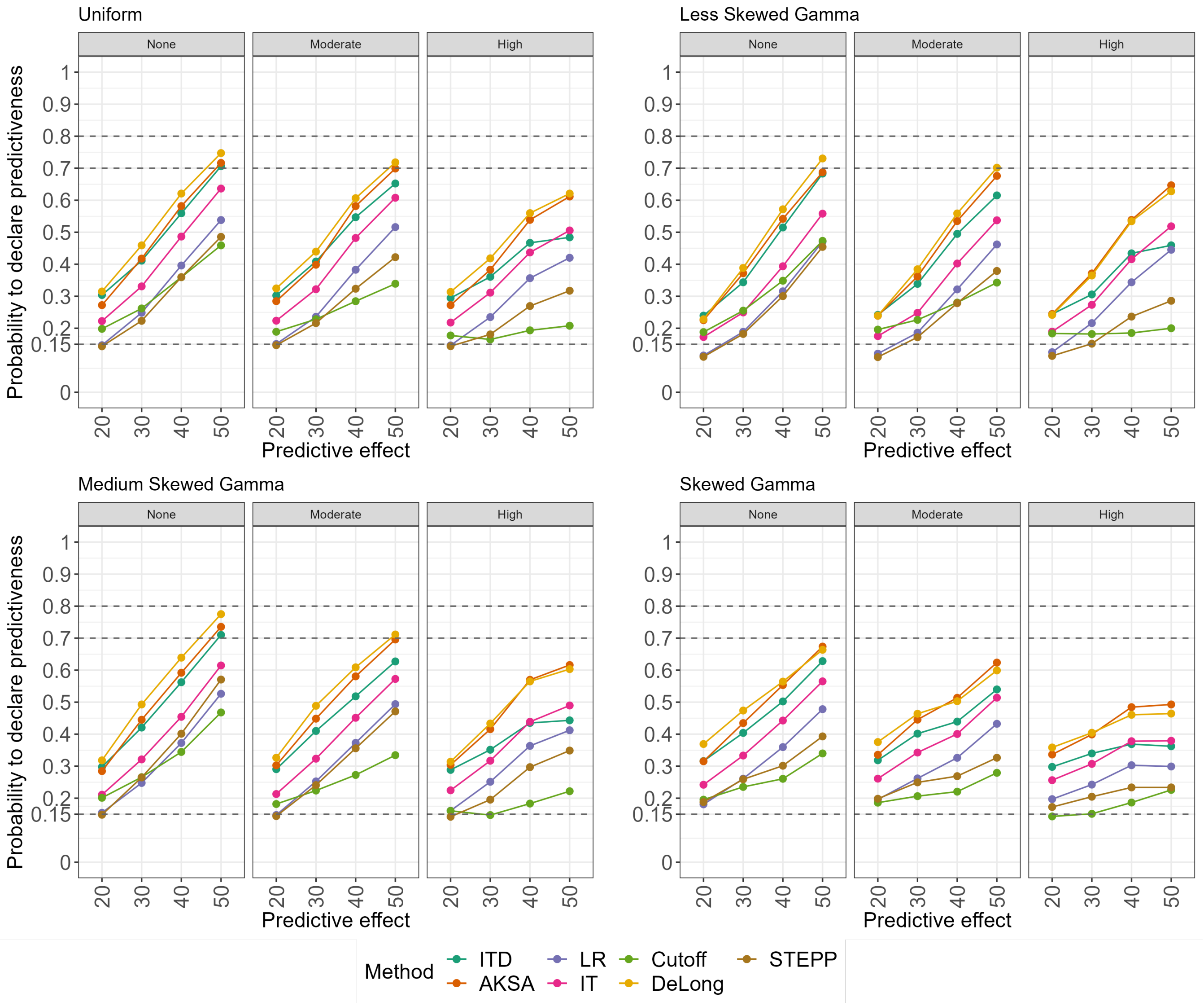

FIGURE 18 Results for all non-null scenarios and for all approaches: interaction test (IT), interaction test with a dichotomous bmk (ITD), likelihood ratio test (LR), STEPP approach (STEPP), probability to find a cutoff (Cutoff), DeLong test (DeLong), difference between two curves (AKSA) for different distributions of the biomarker and when the sample size is 40:40 patients (experimental:control). Columns in the graphs represent the prognostic effect (N = null, M = moderate and H=high). On the x-axis instead the predictive effect is reported (20%, 30%, 40% or 50%). Biomarker-response data are generated following a step function.

4.2.3 | Results with a total sample size of 200 (100:100) patients

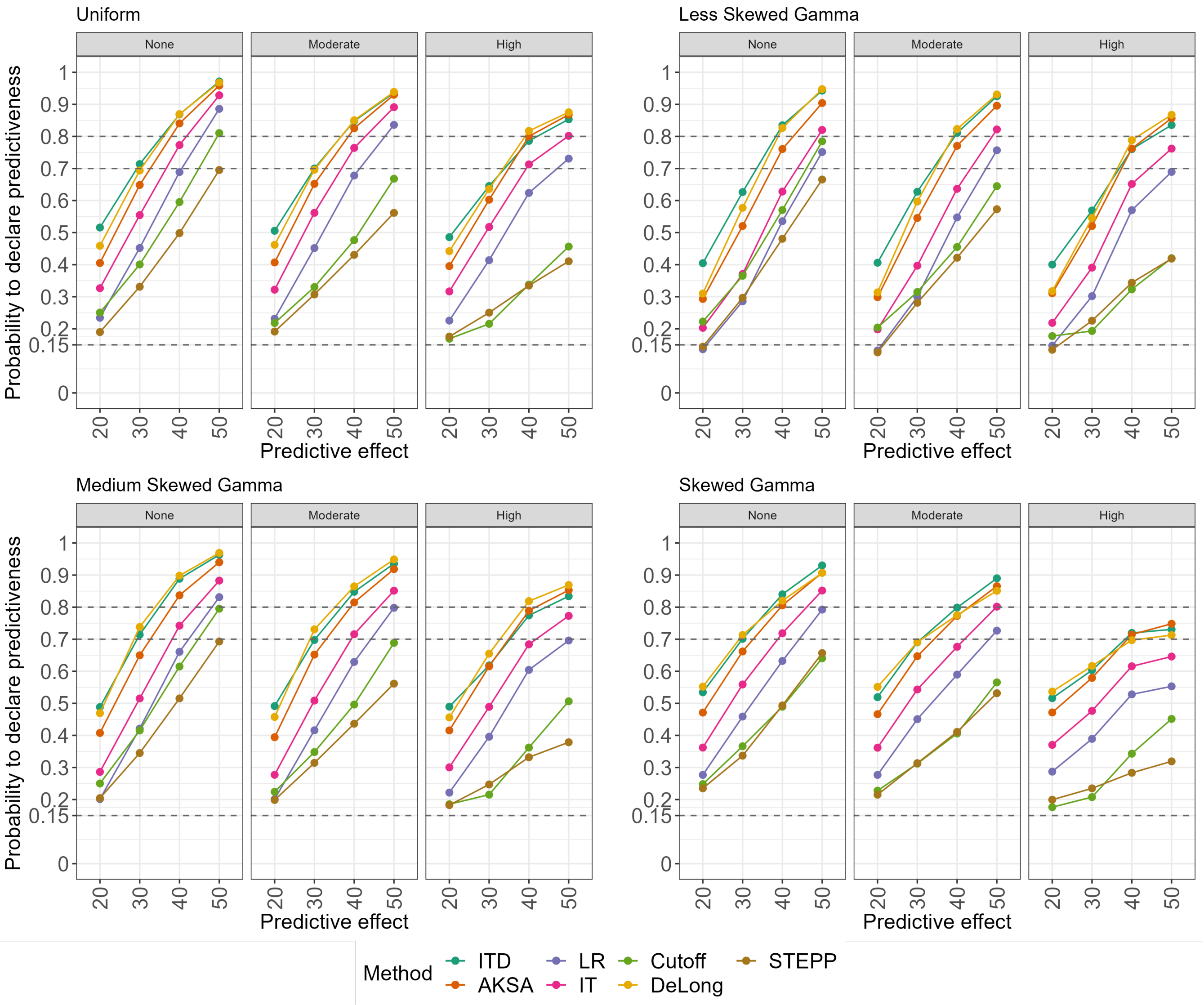

FIGURE 19 Results for all non-null scenarios and for all approaches: interaction test (IT), interaction test with a dichotomous bmk (ITD), likelihood ratio test (LR), STEPP approach (STEPP), probability to find a cutoff (Cutoff), DeLong test (DeLong), difference between two curves (AKSA) for different distributions of the biomarker and when the sample size is 100:100 patients (experimental:control). Columns in the graphs represent the prognostic effect (N = null, M = moderate and H=high). On the x-axis instead the predictive effect is reported (20%, 30%, 40% or 50%). Biomarker-response data are generated following a step function.

5 | QUALITATIVE INTERACTION TEST

A biomarker is predictive if it can be shown that there is an interaction between the treatment effect and the biomarker status. However, the interaction can be of two types: qualitative and quantitative[3]. Quantitative interactions look at whether there is a positive treatment effect in both two biomarker subgroups (e.g. biomarker-positive and negative patients) but the amount of treatment benefit is different for the two subgroups. The qualitative interaction instead looks at whether there is a positive treatment effect in one subgroup and not in the other, that is whether the treatment effects in both subgroups have or not the same sign (e.g. higher response rate in both subgroups or not). Thus, additionally, to the approaches presented in this work, a qualitative interaction test has also been explored[4]. The biomarker space is divided into two subgroups $X > \tilde{m}$ and $X \leq \tilde{m}$ (above and below the estimated median $\tilde{m}$) and for each subgroup, a linear logistic model with only the treatment as a covariate is used to fit the data, that is

- Model 1: $Y^{(k)} \sim T_k$ when $X > \tilde{m}$
- Model 2: $Y^{(k)} \sim T_k$ when $X \leq \tilde{m}$

and the treatment effects $\tilde{\delta}_1$ and $\tilde{\delta}_2$ and their corresponding standard errors se_{δ_1} and se_{δ_2} from Model 1 and Model 2 are estimated. The null hypothesis we are interested in is that there are no crossover interactions. That means

$$H_0 : \delta_1 \leq 0 \cap \delta_2 \leq 0 \;\text{ or }\; H_0 : \delta_1 \geq 0 \cap \delta_2 \geq 0$$

Then the likelihood ratio test of this hypothesis is applied as described in Gail and Simon (1985)[4]. The p-value of the test is then compared to the threshold α_Q (chosen to control the type I error rate at level α). If the p-value is less than α_Q, then the biomarker is declared as predictive.

5.1 | Results for all scenarios for qualitative interaction test

	Total SS 60				Total SS 80				Total SS 200			
	$X \sim U(0,100)$	$X \sim \Gamma(0.083, s)$	$X \sim \Gamma(0.069, s)$	$X \sim \Gamma(0.049, s)$	$X \sim U(0,100)$	$X \sim \Gamma(0.083, s)$	$X \sim \Gamma(0.069, s)$	$X \sim \Gamma(0.049, s)$	$X \sim U(0,100)$	$X \sim \Gamma(0.083, s)$	$X \sim \Gamma(0.069, s)$	$X \sim \Gamma(0.049, s)$
NPNPT	0.06	0.07	0.07	0.06	0.05	0.05	0.05	0.05	0.01	0.01	0.01	0.01
NPPT	0.07	0.06	0.07	0.06	0.05	0.06	0.05	0.06	0.01	0.01	0.01	0.01
NPNPNT	0.13	0.13	0.14	0.14	0.15	0.16	0.17	0.16	0.15	0.15	0.15	0.14
NPPNT	0.12	0.13	0.13	0.11	0.15	0.15	0.16	0.15	0.15	0.15	0.14	0.15
HPNP1_50	0.29	0.16	0.29	0.39	0.33	0.16	0.35	0.53	0.4	0.1	0.4	0.8
HPMP1_50	0.26	0.14	0.26	0.35	0.29	0.15	0.33	0.47	0.4	0.11	0.41	0.75
HPHP1_50	0.13	0.08	0.18	0.29	0.18	0.11	0.24	0.37	0.38	0.13	0.39	0.62
HPNP1_40	0.22	0.14	0.21	0.29	0.25	0.13	0.25	0.37	0.25	0.07	0.25	0.55
HPMP1_40	0.22	0.13	0.2	0.26	0.24	0.12	0.23	0.35	0.24	0.08	0.25	0.53
HPHP1_40	0.15	0.1	0.17	0.25	0.2	0.11	0.21	0.31	0.25	0.08	0.24	0.49
MPNP1_30	0.16	0.11	0.15	0.2	0.18	0.1	0.17	0.27	0.13	0.04	0.12	0.33
MPMP1_30	0.17	0.1	0.15	0.2	0.17	0.1	0.17	0.25	0.13	0.04	0.13	0.31
MPHP1_30	0.14	0.1	0.14	0.2	0.16	0.09	0.14	0.24	0.13	0.05	0.12	0.28
LPNP1_20	0.1	0.08	0.11	0.13	0.1	0.07	0.11	0.15	0.06	0.02	0.05	0.12
LPMP1_20	0.11	0.08	0.1	0.14	0.1	0.07	0.1	0.15	0.05	0.02	0.05	0.11
LPHP1_20	0.1	0.08	0.1	0.13	0.1	0.08	0.1	0.15	0.05	0.02	0.05	0.11
HPNP2_50	0.45	0.32	0.37	0.32	0.58	0.39	0.51	0.41	0.8	0.54	0.78	0.59
HPMP2_50	0.42	0.3	0.35	0.29	0.55	0.39	0.47	0.37	0.8	0.52	0.73	0.48
HPHP2_50	0.28	0.2	0.27	0.23	0.36	0.28	0.36	0.26	0.73	0.5	0.61	0.34
HPNP2_40	0.35	0.25	0.3	0.26	0.46	0.33	0.39	0.36	0.68	0.43	0.63	0.53
HPMP2_40	0.34	0.25	0.28	0.25	0.44	0.31	0.37	0.34	0.66	0.42	0.59	0.47
HPHP2_40	0.29	0.2	0.24	0.23	0.38	0.27	0.34	0.29	0.64	0.41	0.52	0.4
MPNP2_30	0.27	0.19	0.22	0.22	0.33	0.23	0.29	0.29	0.47	0.29	0.4	0.43
MPMP2_30	0.25	0.19	0.22	0.22	0.34	0.24	0.27	0.28	0.45	0.26	0.41	0.42
MPHP2_30	0.24	0.17	0.2	0.21	0.3	0.23	0.26	0.27	0.43	0.27	0.36	0.38
LPNP2_20	0.2	0.15	0.17	0.18	0.24	0.17	0.2	0.23	0.27	0.17	0.24	0.31
LPMP2_20	0.19	0.16	0.17	0.18	0.23	0.18	0.21	0.23	0.27	0.16	0.24	0.31
LPHP2_20	0.19	0.15	0.17	0.19	0.23	0.17	0.2	0.23	0.27	0.16	0.23	0.29

TABLE 8 Results for all scenarios, all distributions of the biomarker and all sample sizes (SS) for the qualitative interaction test where the α_Q = 0.27. Biomarker-response data are generated following a logistic function.

	Total SS 60				Total SS 80				Total SS 200			
	$X \sim U(0,100)$	$X \sim \Gamma(0.083,s)$	$X \sim \Gamma(0.069,s)$	$X \sim \Gamma(0.049,s)$	$X \sim U(0,100)$	$X \sim \Gamma(0.083,s)$	$X \sim \Gamma(0.069,s)$	$X \sim \Gamma(0.049,s)$	$X \sim U(0,100)$	$X \sim \Gamma(0.083,s)$	$X \sim \Gamma(0.069,s)$	$X \sim \Gamma(0.049,s)$
NPNPT	0.06	0.07	0.07	0.06	0.05	0.05	0.05	0.05	0.01	0.01	0.01	0.01
NPPT	0.07	0.07	0.08	0.07	0.06	0.06	0.06	0.06	0.01	0.01	0.01	0.02
NPNPNT	0.14	0.14	0.13	0.14	0.15	0.16	0.15	0.15	0.15	0.14	0.14	0.15
NPPNT	0.15	0.14	0.14	0.13	0.16	0.15	0.15	0.14	0.14	0.14	0.14	0.14
HPNP1_50	0.16	0.06	0.19	0.42	0.18	0.04	0.21	0.55	0.13	0.01	0.17	0.79
HPMP1_50	0.19	0.08	0.21	0.38	0.22	0.07	0.26	0.51	0.22	0.03	0.27	0.77
HPHP1_50	0.17	0.09	0.19	0.27	0.23	0.13	0.24	0.35	0.36	0.13	0.39	0.59
HPNP1_40	0.12	0.05	0.11	0.31	0.11	0.03	0.11	0.41	0.05	0	0.05	0.58
HPMP1_40	0.13	0.07	0.12	0.29	0.15	0.04	0.13	0.39	0.08	0.01	0.09	0.56
HPHP1_40	0.15	0.08	0.13	0.27	0.17	0.08	0.17	0.35	0.16	0.03	0.15	0.57
MPNP1_30	0.07	0.04	0.06	0.14	0.06	0.03	0.05	0.15	0.01	0	0	0.11
MPMP1_30	0.08	0.05	0.07	0.15	0.07	0.04	0.05	0.17	0.02	0	0.01	0.13
MPHP1_30	0.1	0.06	0.08	0.16	0.1	0.06	0.08	0.18	0.05	0.01	0.03	0.17
LPNP1_20	0.06	0.07	0.06	0.07	0.05	0.05	0.05	0.05	0.01	0.01	0.01	0.01
LPMP1_20	0.07	0.06	0.06	0.06	0.05	0.05	0.05	0.05	0.01	0.01	0.01	0.01
LPHP1_20	0.07	0.08	0.08	0.08	0.07	0.07	0.07	0.06	0.01	0.02	0.02	0.02
HPNP2_50	0.36	0.3	0.45	0.26	0.48	0.39	0.6	0.35	0.68	0.47	0.87	0.52
HPMP2_50	0.35	0.3	0.39	0.22	0.47	0.41	0.53	0.29	0.71	0.56	0.84	0.38
HPHP2_50	0.28	0.25	0.3	0.16	0.39	0.33	0.4	0.18	0.69	0.6	0.69	0.19
HPNP2_40	0.29	0.22	0.35	0.23	0.37	0.26	0.47	0.31	0.49	0.31	0.74	0.48
HPMP2_40	0.28	0.22	0.34	0.22	0.36	0.28	0.44	0.27	0.51	0.33	0.71	0.42
HPHP2_40	0.25	0.23	0.3	0.2	0.34	0.27	0.39	0.24	0.53	0.39	0.68	0.31
MPNP2_30	0.18	0.14	0.24	0.23	0.23	0.16	0.31	0.3	0.28	0.12	0.41	0.47
MPMP2_30	0.19	0.15	0.24	0.23	0.23	0.16	0.29	0.29	0.3	0.15	0.42	0.42
MPHP2_30	0.19	0.15	0.21	0.2	0.23	0.18	0.27	0.25	0.31	0.18	0.4	0.36
LPNP2_20	0.14	0.11	0.14	0.19	0.15	0.11	0.17	0.25	0.14	0.06	0.14	0.35
LPMP2_20	0.15	0.12	0.15	0.2	0.16	0.11	0.16	0.26	0.13	0.06	0.14	0.35
LPHP2_20	0.15	0.11	0.15	0.18	0.16	0.12	0.16	0.25	0.13	0.06	0.15	0.34

TABLE 9 Results for all scenarios, all distributions of the biomarker and all sample sizes (SS) for the qualitative interaction test where the $\alpha_Q = 0.27$. Biomarker-response data are generated following a step function.

ACKNOWLEDGMENTS

This work was supported by Institut de Recherches Internationales Servier. The results reported herein are part of a collaboration between Servier, Saryga, and P Mozgunov and A Serra, whose research is supported by the National Institute for Health and Care Research (NIHR Advanced Fellowship, Dr Pavel Mozgunov, NIHR300576). The views expressed in this publication are those of the authors and not necessarily those of the NHS, the National Institute for Health and Care Research or the Department of Health and Social Care (DHCS). P Mozgunov and A Serra received funding from UK Medical Research Council (MC UU 00040/03). For the purpose of open access, the author has applied a Creative Commons Attribution (CC BY) license to any author accepted manuscript version arising

Author contributions

All authors have directly participated in the planning and execution of the presented work.

Financial disclosure

None reported.

Conflict of interest

JG and SG are the employees of Institut de Recherches Internationales Servier. GSH is President of Saryga SAS. MKR and HH are employees of Saryga SAS. PM and AS served as statistical consultants for Institut de Recherches Internationales Servier and Saryga SAS.

SUPPORTING INFORMATION

Additional supporting information may be found online in the Supporting Information section at the end of this article.

REFERENCES

1. Agresti A. *Categorical data analysis*. 792. John Wiley & Sons, 2002.
2. Casella G, Berger R. *Statistical inference*. CRC Press, 2024.
3. Polley MYC, Freidlin B, Korn EL, Conley BA, Abrams JS, McShane LM. Statistical and practical considerations for clinical evaluation of predictive biomarkers. *Journal of the National Cancer Institute.* 2013;105(22):1677–1683.
4. Gail M, Simon R. Testing for qualitative interactions between treatment effects and patient subsets. *Biometrics.* 1985:361–372.
